# Peer Victimization in Childhood and Timing of Substance Use Initiation: Evidence from a Twin Study

**DOI:** 10.1007/s10519-025-10222-4

**Published:** 2025-07-12

**Authors:** Li Hazel Yu, Kristine Marceau, Valerie S. Knopik, Laura Baker

**Affiliations:** 1 Department of Human Development and Family Science, Purdue University, West Lafayette, IN USA; 2 Department of Psychology, University of Southern California, Los Angeles, CA USA

**Keywords:** Substance use initiation, Peer victimization, Twin modeling, Childhood, Adolescence

## Abstract

Previous studies robustly link childhood peer victimization experience to the timing of substance use initiation. However, no study has investigated the contributions of genetic and environmental factors to this link. The current study focused on a sample of 779 twin pairs followed from age 9–10 to 19–20, which is racially/ethnically and socioeconomically representative of the greater Los Angeles area. The aims were to investigate (1) the associations between childhood victimization, including physical (e.g., kicking, pushing), verbal (e.g., taunting), and relational victimization (e.g., spreading rumors), and timing of substance use initiation, and (2) the contributions of genetic/environmental factors to these associations. Multinomial logistic regressions revealed several small associations, but none of these survived corrections for multiple testing. Univariate genetic models suggested genetic (A) and nonshared environmental influences (E) on verbal victimization (V_A_ = .43, V_E_ = .57), shared environmental (C) and nonshared environmental factors on relational victimization (V_C_ = .22, V_E_ = .78), and ambiguous familial influences and E on physical victimization (V_A_ = .34, V_E_ = .66; V_C_ = .26, V_E_ = .74). Timing of cigarette initiation were explained by A, C, and E (V_A_ = .48, V_C_ = .31, V_E_ = .21). Quantitative sex differences in contributions of A, C, and E were detected for alcohol (V_AM_ = .90, V_EM_ = .10; V_CF_ = .86, V_EF_ = .14) and marijuana initiation (V_AM_ = .89, V_EM_ = .11; V_CF_ = .79, V_EF_ = .21); however, A could be dropped for females and C could be dropped for males across both variables. Multivariate twin analyses were not feasible, due to the low cross-trait correlations. These findings call into question the robustness of links between self-reported victimization in childhood and prospectively measured timing of substance initiation across adolescence.

**Supplementary Information:**

The online version contains supplementary material available at 10.1007/s10519-025-10222-4.

## Introduction

Peer victimization, also known as bullying victimization, is characterized by experiences including being beaten up, threatened, humiliated, and intentionally excluded (Sapouna & Wolke 2013). In the United States, 10–35% of primary school students report encountering peer victimization (Biswas et al. 2020; Kshirsagar et al. 2007). These experiences often occur repeatedly over time and between individuals or groups with differences in social status or power, where the victim finds themselves powerless to defend against the perpetrator (Monks et al. 2009; Sapouna & Wolke 2013). These occurrences manifest across various contexts, including offline contexts (e.g., schools, neighborhoods, workplaces) and online settings, spanning diverse cultural landscapes (Kowalski et al. 2019; Schumann et al. 2014).

In offline contexts, peer victimization can manifest in various, highly correlated forms, including physical, verbal, and relational victimization. Physical victimization is characterized by physically hurting the victim, including hitting, kicking, or pushing the individual (Wang et al. 2009). Verbal victimization commonly refers to the use of hurtful names, taunting, and derisive comments directed at the victim (Wang et al. 2009). Relational victimization, or indirect victimization/covert victimization, involves actions such as social exclusion from peer groups and the dissemination of malicious rumors targeting the victim (Casper & Card 2017).

The prevalence and developmental trajectory of victimization vary across specific forms. Physical victimization typically emerges in kindergarten years, reaches its peak in elementary school, and decreases during adolescence (Scheithauer et al. 2006; Wang et al. 2009), when individuals acquire and turn to more sophisticated, covert strategies to show malice (Crick & Grotpeter 1995). Developmentally sequenced, verbal and relational victimization are found to reach the peak during middle school years, and even persist until high school (Smith & Gross 2006). In a nationally representative sample, Wang et al. (2009) found that 12.8% of middle school students in the United States had encountered physical victimization within the past two months, whereas the rates of verbal and relational victimization were 36.5% and 41.0%, respectively. When children aged 0 to 17 are grouped together, the prevalence of physical victimization is 37.3%, while relational victimization stands at 36% (Finkelhor et al. 2015; Gladden et al. 2014).

A substantial body of research indicates that peer victimization constitutes an adverse event with harmful consequences for victims in both short- and long-term timescales (Klomek et al. 2015). Those subjected to victimization tend to experience psychological distress, exhibiting higher social withdrawal, and displaying aggression toward others (Brendgen et al. 2008; Earnshaw et al. 2017; Wei & Chen 2008). Some negative consequences, such as poor self-esteem, elevated loneliness and depressive symptoms, may persist until late adolescence and, in some cases, even extend into adulthood (Matthews et al. 2022; McDougall & Vaillancourt 2015; Smithyman et al. 2014; Storch & Ledley 2005).

## The Link Between Peer Victimization and Substance Use

One of the consequences of peer victimization is heightened risk for substance use, particularly problematic use of alcohol, marijuana, and other illicit drugs (Earnshaw et al. 2017; Sullivan et al. 2006). In a cross-sectional study, the experience of peer victimization in the past 30 days predicted alcohol, tobacco, marijuana, and illicit drug use in modest magnitudes (Doumas et al. 2017). Rospenda and colleagues (2013) found that work and school bullying victimization in college increased alcohol use in small to modest magnitudes. Moreover, Connolly (2017) reported that developmental trajectories of alcohol, cigarettes, and marijuana use from early adolescence to middle adulthood differed as a function of being bullied as a child. Specifically, among boys who experienced bullying, there was a trajectory of earlier initiation and slower decline in marijuana and cigarette use, compared with non-victims. Victimized girls, compared with non-victimized girls, exhibited a distinct pattern, with a faster uptake of cigarette use and a slower decline in both alcohol and cigarette use. Moreover, both cross-sectional and longitudinal studies focusing on 10- to 15-year-old adolescents have revealed that victims of peer bullying tend to engage in underage use of alcohol, cigarettes, and marijuana, in small but significant magnitudes (Davis et al. 2018; Laroque et al. 2023; Walters & Espelage 2018). These previous findings warrant a comprehensive exploration, forming the basis of the present study.

Past evidence also suggests the possibility that specific forms of victimization are associated with different types of substance use, or the same type of substance but in different magnitudes. One cross-sectional study focusing on African American adolescents in 8th grade showed that physical victimization was moderately associated with alcohol use, whereas relational victimization was more strongly linked to marijuana use (Sullivan et al. 2006). In another cross-sectional study on Italian secondary school students aged 13–15, physical and relational victimization was associated with smoking and drinking in medium to large magnitudes, but was not linked to verbal victimization (Vieno et al. 2011). Thus, the current study tested the association of timing of initiation of each type of substance (smoking, drinking, and marijuana) with each form of peer victimization (relational, verbal, and physical), to further elucidate possible impacts of distinct victimization experiences.

Regarding elucidating the mechanisms underlying the association of peer victimization and substance use, several explanations have been proposed, focusing on psychosocial and biological factors, and interplay between individual attributes and contextual factors. The first dominant psychosocial explanation encompasses the stress-coping and self-medication hypotheses, both suggesting that individuals subjected to peer victimization possibly resort to drug use to cope with psychological stress induced by the exposure to victimization (Khantzian 1985, 1997). Specifically, youth who encounter peer victimization may experience anguished feelings, such as frustration and resentment (Cañas et al. 2022), and therefore turn to substance use as a means of alleviating emotional distress and maintaining a sense of emotional equilibrium. Another explanation posits that the experience of victimization renders the victim more susceptible to environmental influences, such as impacts from deviant peers (Jiang et al. 2016). Prior research has demonstrated that affiliating with deviant peer groups is a robust predictor of substance use in adolescence and young adulthood (Fergusson et al. 2002). Adolescents often engage in substance use in the presence of peers to conform and gain social acceptance from peers (Van Ryzin & Dishion 2014), whereas deviant peer groups are more inclined to provide access to drugs (Fergusson et al. 2002), which serves as an external influence on adolescents’ substance use. Past research has indicated that victimized children and adolescents are more likely to be excluded from mainstream peer groups, potentially leading them toward marginalized, deviant peers (Rudolph et al. 2014; Veenstra et al. 2005) which may increase the risk of using drugs.

These prevailing explanations shed light on the environmental and psychological mechanisms that may account for the association between peer victimization and substance use. However, an alternative explanation involving a third variable remains plausible. That is, the exploration of whether genetic factors contribute to individual differences in this association is still lacking. Tackling whether there are any common genetic factors underlying the association of peer victimization and substance use initiation can not only elucidate the etiology of the co-occurrence of both constructs but also help to identify whether peer victimization indeed exerts an environmental influence on substance use, as posited by these theoretical explanations.

Despite the abundance of research investigating the association of early-life peer victimization and underage substance use, much of the existing evidence is derived from cross-sectional or short-term longitudinal data and often neglects the control for genetic and/or environmental influences. Consequently, less is known about the extent to which genetic effects explain individual differences in the association between peer victimization and the timing of substance initiation. Leveraging genetically informed designs, however, enables researchers to disentangle these unique effects, and shed new light on the underlying bio-psycho-social factors that contribute to heightened risks of both victimization and substance initiation. As such, the present study aims to investigate the extent to which the association between early childhood peer victimization and timing of substance use initiation is accounted for by shared genetic and/or environmental influences.

## Genetic and Environmental Contributions to the Link Between Peer Victimization and Substance Use Initiation

It is possible that peer victimization and initiation of substance use could be linked via common genetic influences underlying the two constructs. Prior genetically informed studies yielded modest to substantial heritability of tobacco (*h*^*2*^ = 26%–60%), alcohol (*h*^*2*^ = 39%–55%), and marijuana initiation (*h*^*2*^ = 35%–72%) (Fowler et al. 2007; Rhee et al. 2003; Shelton et al. 2007). The remaining proportions of individual differences are accounted for by both shared and nonshared environmental influences.

It is important to note that the evidence of potential genetic influences on peer victimization is currently muddied by conflicting findings. Research based on twin data shows that the heritability of peer victimization is 32% in preschool children (DiLalla & John 2014), 62%–70% in children aged 9.48 on average (Veldkamp et al. 2019), and 73% in children aged 10 (Ball et al. 2008). In one study focusing on 6-year-old children, however, peer victimization was suggested to be purely environmentally driven, with no significant genetic influences (Brendgen et al. 2008).

This inconsistency in findings is possibly due to the variations in the populations recruited and/or the measures employed. Higher heritability of peer victimization is generally detected among older children and adolescents than in younger children, or when using self-report or mother’s report than when using peer nominations. This age difference may reflect an active gene-environment correlation (Scarr & McCartney 1983), wherein adolescents are more able to select their environments, and may choose environments consistent with their genetic predispositions that allow them to use substances but also come with increased risk of victimization (e.g., antisocial friend groups and/or unsupervised situations).

Several scholars have argued that peer victimization is an exposure rather than a direct behavior (Veldkamp et al. 2019), that estimates of genetic and environmental influences may actually reflect the heritable and environmental characteristics that impact the child’s susceptibility to victimization. Traits such as social deficits, shyness, impulsivity, and emotional dysregulation are known to influence an individual’s vulnerability to victimization (Ball et al. 2008; Mynard & Joseph 1997). Importantly, previous research has also revealed that these personality and behavioral characteristics also share genetic and environmental influences with substance use and initiation (Z. Chang et al. 2012; Hicks et al. 2014). Consequently, interpretations concerning genetic and environmental influences on peer victimization, as well as the genetic and environmental overlap with substance initiation, require careful consideration.

## The Present Study

To address the gaps in the literature, the present study employed a genetically informed longitudinal twin design to examine the etiological overlap between peer victimization and adolescents’ timing of substance use initiation. Our first aim was to address phenotypic associations between peer victimization and timing of substance use. According to previous evidence (e.g., Tharp-Taylor et al. 2009), we hypothesized that peer victimization at age 9–10 predicts earlier timing of substance use initiation. Specifically, this prediction pathway was tested separately for alcohol, cigarette, and marijuana. Participants’ age, sex, and race/ethnicity were included as covariates. Our second aim was to examine the genetic and environmental contributions to these phenotypic associations. Given the scarcity of former research focusing on the genetic and environmental contributions to this link, no specific hypothesis concerning the relative contributions was posited.

## Method

### Participants

Data for the present study were drawn from the University of Southern California (USC) longitudinal study of Risk Factors for Antisocial Behavior (RFAB) (Baker et al. 2006, 2013). Participants were 780 sets of twins and triplets that were tracked from ages 9–10, with 779 twin pairs (N = 770 pairs) or triplets (N = 9 triplets) provided available data for analyses, and one twin pair was removed because no data were available. From each set of triplets, two children were randomly selected for analysis. Sex composition was approximately equal, comprising same-sex monozygotic (MZ) and dizygotic (DZ) pairs, as well as opposite-sex DZ pairs. The study includes five waves of data collection in total, occurring every 2–3 years, until ages 19–20 (Wave 1: age 9–10 years, 614 pairs; Wave 2: age 11–13 years, 445 pairs; Wave 3: age 14–15 years, 604 pairs; Wave 4: age 16–18 years, 504 pairs; and Wave 5: age 19–20 years, 625 pairs). The sample was representative of the racial/ethnic and socioeconomic diversity of the greater Los Angeles area, including 34.3% Hispanic Americans, 28.6% Caucasian Americans, 17.6% Multiracial, 13.1% Black/African Americans, 4.1% Asian Americans, 2.2% other or unknown, and 0.1% Native Americans.

### Procedures

Beginning in 2000, the Southern California Twin Register expanded to include a sample of school-aged twins/triplets and their families, recruited through advertisements, schools, and mothers of twins clubs, which composed the RFAB sample (Baker et al. 2006, 2013). The twins/triplets were born in 1990–1995. The inclusion criteria were: (1) the age of twins/triplets were 9–10 years old at the time of the first wave; (2) the twins/triplets were fluent in English; (3) participants were available to complete a 6–8-h laboratory assessment at USC; and (4) the primary caregiver was fluent in either English or Spanish.

### Measures

### Childhood Peer Victimization

Children self-reported on the four-item Childhood Peer Victimization subscale in the Child Friendship Questionnaire (CFQ), which was specifically developed for the RFAB. One item was for physical victimization (“How often does someone at school hit, push, or start fights with you?”), two items were for verbal victimization (e.g., “How often does someone at school tease or make fun of you?”), and one item was for relational victimization (“How often does someone at school tell you that you can’t play with them or join their group?”). This questionnaire applies a 5-point Likert scale, with 1 = “Never” and 5 = “Almost Always”. Scores were averaged for physical, verbal, and relational victimization. Current study utilized data collected in Wave 1, when the twins were 9–10 years of age. The Cronbach’s alpha across all items was 0.94.

### Child/adolescent Timing of Substance Use Initiation

The timing of substance use initiation in the twins was assessed in and calculated from all five waves. In each wave of data collection, children reported on three items: (1) *Have you ever had a whole drink or more of alcohol*; (2) *Have you ever smoked*; and (3) *Have you ever tried marijuana*. Timing of substance use initiation was calculated based on the wave that the twins reported the first ever use. Four levels of timing of substance use initiation were created for each drug: 4 = Early Initiation: if the child/adolescent reported the first ever use at or before age 15 (Wave 3); 3 = Mid Initiation: if the child/adolescent reported the first ever use at age 16–18 (Wave 4); 2 = Late Initiation: if the child/adolescent reported the first ever use at age 19–20 (Wave 5); and 1 = Never Endorsed by the Final Assessment: if the child/adolescent never reported ever use in any wave. Thus, higher scores indicate earlier initiation. Due to the extremely rare cases of substance initiation in Wave 1 and 2, the early initiation level incorporated initiation in Wave 1 through 3 (see *Supplementary Material S1*).

### Covariates

Covariates included in the present study were the twins’ age, sex, and dummy-coded race/ethnicity.

## Missing Data

In total, 779 twin pairs or triplets provided any available data in any waves of data collection. However, only 237 twin pairs had complete data (at least one victimization variable, at least one substance initiation variable, and covariate data) for both twins. This discrepancy in sample size is due mainly to the addition of > 200 twin pairs after wave 2 who do not have measures of peer victimization from wave 1. We examined Little’s missing completely at random (MCAR) test and compared those with complete data to the full sample using generalized linear models (GLM) (Little 1988). Results revealed that the data were not missing completely at random, *χ*^*2*^(55) = 100, *p* < 0.001. GLM showed that participants who were younger at baseline were more likely to have missing data on timing of alcohol use initiation. Male and non-Caucasian participants were more likely to have missing reports on timing of cigarette use initiation. Male, younger participants were more likely to have missingness on timing of marijuana use initiation. Thus, for the main analysis, we use the full sample and Full Information Maximum Likelihood estimation to maximize power and representativeness of the sample.

## Analytic Plan

Data analyses were performed using SAS 9.4, OpenMx in R (Hunter 2018; Neale et al. 2016), and STATA (Hamilton & Seyfrit 1993). As described below, our analysis plan included phenotypic analyses and biometric modeling. Phenotypic analyses included preliminary descriptive analyses, mixed-effect models to test sex and racial/ethnic differences while controlling for the nested data structure, and multinomial logistic regression to address our first research aim. Biometric modeling analyses included preliminary cross-twin within-trait and cross-trait correlations, tests of sex differences, and univariate decompositions. Provided sufficient correlations between constructs (e.g., *r* > 0.20; Keskitalo et al. 2007; Tabachnick & Fidell 2018), multivariate twin modeling (Cholesky and common factor analyses) was planned to address our second research aim. These steps were replicated in sensitivity analysis, in the sample 237 twin pairs or triplets with (1) at least one peer victimization variable, and (2) at least one timing of substance use initiation variable.

### Phenotypic Analyses

First, descriptive analyses were conducted to check the distributional properties of the six main variables. Any skewed variables (skewness >  + 1.0 or < − 1.0) were then transformed as needed to correct the skewness. Second, mixed-effect models were run to test any sex differences in peer victimization and substance initiation variables, while controlling for the data structure that children are nested within families. Prior research has detected sex differences in all variables (Carbone-Lopez et al. 2010; Wang et al. 2009). In U.S., boys report experiencing more physical victimization, usually initiate drinking, smoking, and marijuana use earlier, whereas girls are more likely to be the target of verbal as well as relational bullying (Carbone-Lopez et al. 2010; Gajos et al. 2023; Morales et al. 2019; Wang et al. 2009; Zilberman et al. 2004), though decreasing trends in sex differences have been observed in the past two decades (Boulton et al. 2002; Keyes et al. 2010). For racial/ethnic differences, previous studies revealed that Black/African and Hispanic children are more likely to be victimized at school than Caucasian American children, while Asian American children report less peer victimization than all other groups (Hanish & Guerra 2000; Sapouna & Wolke 2013). For timing of substance use initiation, Caucasian American youths tend to initiate all three types of drugs earlier than other groups, and Asian American youths are usually likely to report the latest initiation (Guerra et al. 2000; Wu et al. 2010).

Because our timing of initiation variable is an ordinal variable and threshold effects are possible, to investigate the first aim, multinomial logistic regression was performed for all three forms of peer victimization in predicting timing of initiation of each type of substance, respectively. The adolescent twins’ age, sex, and six dummy-coded race/ethnicity variables (i.e., Hispanic, Black/African, Asian, Native American, Multiracial, Unknown; the Caucasian American group was left out as the reference group) entered the models as covariates. To control for the nested structure of our data, the Huber-White sandwich estimator was used (Huber 1967; White 1980).

### Biometric Modeling

We ran univariate decompositions to capture the contributions of genetic factors (A), shared environmental factors (C) or dominant genetic factors (D), and nonshared environmental factors (E) for all six variables, based on indications from the cross-twin within-trait correlations. For the biometric modeling portion of this paper, we treated the ordinal outcome variable as continuous to reduce model complexity. This is a reasonable approximation of our ordinally measured timing of initiation variable when considering the timing of substance use initiation as an index of severity (i.e., considering earlier onset as a linearly more severe phenotype), corroborated with prior evidence from longitudinal studies showing earlier initiation is linked to increased likelihood of reaching more severe phenotypes, and studies showing impacts of substance use on the adolescent brain (Jackson et al. 2021; Lees et al. 2020; Thorpe et al. 2020).

Because MZ twins share 100% of their segregating genes and DZ twins share on average 50%, comparing phenotypic correlations of these two types of twins provides a rationale for decomposing the variance of the phenotypic variable into A, C (or D), and E. An important preliminary step for conducting biometric modeling is to test cross-twin within-trait and cross-trait correlations, which show the pattern of correlations within and across traits in MZ and DZ twins. Results from these correlations inform the type of univariate twin modeling – particularly whether a C or a D should be included, given that univariate models using only MZ and DZ twins are not identified to estimate both simultaneously. C is indicated when DZ correlations are higher than half the size of MZ correlations, whereas D is indicated when DZ correlations are less than half the size of MZ correlations.

Then we ran sex limitation models for each main variable to examine the potential quantitative and qualitative sex differences in the contributions of A, C/D, and E. We firstly specified a full ACE/ADE model that allowed for the estimation of all genetic and environmental influences on the variable without any constraints. Hereafter, we followed the order of (1) fixing the genetic correlation between co-twins to 0.5 in dizygotic opposite-sex twins; (2) fixing the paths of A, C/D, and E to be the same for male and female co-twins in dizygotic opposite-sex twins; and (3) fixing the paths as done in both steps 1 and 2. Non-significant difference from the full ACE/ADE model represented that the contributions of A, C/D, and E did not differ between sex groups.

Nested sub-models, with one or two genetic or shared environmental effects being dropped from the full ACE/ADE model, resulting in AE, CE, and E models. We used standard Chi-square difference tests to determine whether any sub-models revealed more parsimonious fit alternatives to the full ACE/ADE model. Participant’s age and six dummy-coded race/ethnicity variables (i.e., the Caucasian American group was used as the reference group) entered univariate models as covariates.

### Multivariate Twin Analyses

Finally, to address our second aim, we proposed to conduct Cholesky decompositions and common factor analysis to test the relative contributions of A, C/D, and E to the association among peer victimization variables and timing of substance use initiation variables. The Cholesky model decomposes variance into genetic and environmental contributions specific to each variable and shared between variables. The bivariate models would be fitted first, and then we would run the six-variable multivariate decomposition if possible. The specific structure of the multivariate model is eventually determined based on the cross-twin cross-trait correlations.

## Results

### Phenotypic Analyses

#### Sociodemographic Characteristics and Descriptive Results

The mean age of participants was 9.60 (Range = 8–11, SD = 0.59) in Wave 1 and was 19.56 (Range = 17–23, SD = 1.12) in Wave 5. Distributions regarding participants’ sex, ethnicity, and zygosity are shown in Table 1. The frequency of participants reporting that they experienced vs. never experienced each form of victimization is shown in Table 2. Based on descriptive results for all six main variables, only childhood physical victimization was significantly skewed (skewness = 1.61). Given that the mean ± 3SD winsorizing would result in values beyond the range of data, we performed the Box Cox transformation to correct the skewness of this variable. The skewness of childhood physical victimization decreased to 0.68 after transformation. Descriptive statistics are shown in Table 3.

**Table 1:** Sociodemographic Characteristics of Twins

Characteristic	*N*	*%*
Sex		
Female	779	50.1
Male	777	49.9
Race/Ethnicity		
Caucasian	446	28.6
Hispanic	536	34.4
Black	206	13.2
Asian	64	4.1
Native American	2	0.1
Multiracial	276	17.7
Unknown	28	1.8
Zygosity		
MZ males	169 (pairs)	21.6
MZ females	171 (pairs)	21.9
DZ males	121 (pairs)	15.5
DZ females	120 (pairs)	15.3
DZ opposite sex	200 (pairs)	25.6

**Table 2:** Frequency of Participants Reporting Different Levels of Victimization

Form of victimization	Response = 1 (“Never”)		Response = 2 or higher	
	Frequency	%	Frequency	%
Physical victimization	745	64.8	405	35.2
Verbal victimization	358	31.1	793	68.9
Relational victimization	531	46.2	619	53.8

**Table 3:** Descriptive Results for Peer Victimization and Timing of Substance Initiation

Variable	*N*	*M*	*SD*		Level									
				Skewness	1		2			3		4		
					*N*	*%*		*N*	*%*	*N*	*%*		*N*	*%*
Physical victimization	1142	1.72	1.19	1.61	/	/		/	/	/	/		/	/
Verbal victimization	1143	2.17	1.17	.83	/	/		/	/	/	/		/	/
Relational victimization	1142	2.13	1.32	.92	/	/		/	/	/	/		/	/
Timing of cigarette use initiation	930	/	/	.83	563	60.5		74	8.0	120	12.9		173	18.6
Timing of alcohol use initiation	602	/	/	.39	261	43.4		106	17.6	80	13.3		155	25.7
Timing of marijuana use initiation	846	/	/	.82	487	57.6		95	11.2	145	17.1		119	14.1

Note: Due to data skewness, physical victimization was Box-Cox transformed, with the skewness after transformation being .68. For timing of substance use initiation variables, Level 1 = Never endorsed by the final assessment: if the child/adolescent never reported ever use in any wave; Level 2 = Late initiation: if the child/adolescent reported the first ever use at age 19–20 (wave 5); Level 3 = Mid initiation: if the child/adolescent reported the first ever use at age 16–18 (wave 4); Level 4 = Early initiation: if the child/adolescent reported the first ever use at or before age 15 (wave 1–3)

#### Mixed-Effect Model Results

Mixed-effect models revealed that boys reported more physical victimization than girls, while no significant sex differences were detected for other variables (Table 4). Both physical [*F*(6, 548) = 2.98, *p* = 0.01] and verbal victimization [*F*(6, 549) = 2.12, *p* = 0.05] showed racial/ethnic differences, but not relational victimization [*F*(6, 548) = 0.60, *p* = 0.73]. Tukey’s HSD Test for multiple comparison revealed that Black children reported more physical and verbal victimization than Caucasian children (physical: *p* = 0.01, *95% C.I.* = [0.14, 0.53]; verbal: *p* = 0.04, *95% C.I.* = [0.11, 0.50]) (Table 5).

**Table 4:** Mixed-Effect Model Results for Sex Differences in Peer Victimization and Timing of Substance Use Initiation

		*N*	*Mean*	*SD*	*F*	*p*
Physical Victimization	Male	549	1.83	1.23	7.95	.01
	Female	591	1.62	1.13		
Verbal victimization	Male	548	2.16	1.16	.29	.59
	Female	593	2.19	1.18		
Relational victimization	Male	548	2.16	1.33	.95	.33
	Female	592	2.10	1.31		
Timing of cigarette use initiation	Male	435	1.97	1.24	1.20	.27
	Female	495	1.83	1.19		
Timing of alcohol use initiation	Male	289	2.20	1.24	.25	.62
	Female	313	2.22	1.25		
Timing of marijuana use initiation	Male	395	1.94	1.17	1.61	.20
	Female	451	1.82	1.11

**Table 5:** Mixed-Effect Model Results for Racial/Ethnic Differences in Peer Victimization and Timing of Substance Use Initiation

Variable	*F* (df1, df2)	*p*-value	Post Hoc Comparisons
Physical victimization	2.98 (6, 548)	.01	Black > Caucasian (M diff = .34, *p* = .01)
Verbal Victimization	2.12 (6, 549)	.05	Black > Caucasian (M diff = .30, *p* = .04)
Relational Victimization	.60 (6, 548)	.73	/
Timing of cigarette use initiation	6.01 (5, 400)	< .001	Black > Caucasian (M diff = .41, *p* = .01) Black > Latino (M diff = .55, *p* = .01) Black > Asian (M diff = 1.05, *p* < .001) Black > Multiracial (M diff = .42, *p* = .04)
Timing of alcohol use initiation	6.45 (5, 194)	< .001	Caucasian > Latino (M diff = .78, *p* = .001) Caucasian > Asian (M diff = 1.03, *p* = .01) Black > Latino (M diff = .78, *p* = .001) Black > Asian (M diff = 1.03, *p* = .01)
Timing of marijuana use initiation	2.57 (5, 352)	.03	Black > Asian (M diff = .80, *p* = .02)

Note: M diff = difference between means. Higher score on victimization represents more victimization experiences at age 9–10. Higher score on timing of substance use initiation represents earlier initiation

All substance use initiation variables showed significant racial/ethnic differences [cigarette: *F*(5, 400) = 6.01, *p* < 0.001; alcohol: *F*(5, 194) = 6.45, *p* < 0.001; marijuana: *F*(5, 352) = 2.57, *p* = 0.03]. Tukey’s HSD Test demonstrated that Black adolescents engaged in smoking earlier than Caucasian (*p* = 0.01, *95% C.I.* = [0.18, 0.64]), Hispanic (*p* = 0.01, *95% C.I.* = [0.24, 0.86]), Asian (*p* < 0.001, *95% C.I.* = [0.55, 1.55]), and Multiracial youth (*p* = 0.04, *95% C.I.* = [0.14, 0.70]). Caucasian adolescents engaged in alcohol use earlier than Hispanic (*p* = 0.001, *95% C.I.* = [0.39, 1.17]) and Asian adolescents (*p* = 0.01, *95% C.I.* = [0.45, 1.61]), and Black adolescents engaged in alcohol use earlier than Hispanic (*p* = 0.001, *95% C.I.* = [0.40, 1.17]) and Asian youth (*p* = 0.01, *95% C.I.* = [0.46, 1.61]). Asian adolescents used marijuana later than Black adolescents (*p* = 0.02, *95% C.I.* = [0.55, 1.55]).

### Addressing Aim 1: Multinomial Logistic Regression Results

Prior to the multinomial logistic regressions, we visually examined bar charts to understand the timing of substance initiation categories of children who experienced each form of victimization or not (never = 0; rarely/sometimes/often/almost always = 1; see *Supplementary Material* S2). Note this operationalization of 0/1 was used only for visual demonstration of trends, not analytic purposes.

Multinomial logistic regression results are shown in Table 6. Adolescents who encountered more physical victimization tended to initiate cigarette use at or before age 15, compared to those who never used by the final assessment (*RRR* = 4.24, *95%CI* [1.35, 13.28], *p* = 0.01). Adolescents who experienced more verbal victimization were more likely to smoke cigarettes at or before age 15, compared to those who initiated at age 19–20 (*RRR* = 1.65, *95%CI* [1.09, 2.51], *p* = 0.02), or never endorsed (*RRR* = 1.31, *95%CI* [1.04, 1.64], *p* = 0.02). Regarding the initiation timing of alcohol use, adolescents who experienced more physical victimization initiated alcohol use at age 16–18, compared with never endorsed at all (*RRR* = 6.00, *95%CI* [1.08, 33.35], *p* = 0.04). Relational victimization was related to higher probability of initiating marijuana use by age 15, compared with initiation at age 16–18 (*RRR* = 4.98, *95%CI* [1.00, 24.73], *p* = 0.05) and never used at all (*RRR* = 4.40, *95%CI* [1.09, 17.75], *p* = 0.04). It is important to note that only 9 of the 54 possible contrasts across all types of substance initiation had a *p-*value of < 0.05, and none survived Bonferroni, Holm-Bonferroni, or Benjamini–Hochberg corrections, for multiple testing.

**Table 6:** Multinomial Logistic Regressions Between Childhood Victimization and Timing of Substance Use Initiation

(Reference Group = Never Endorsed by the Final Assessment)							
		Physical Victimization		Verbal Victimization		Relational Victimization	
		*RRR (95% CI)*	*p*	*RRR (95% CI)*	*p*	*RRR (95% CI)*	*p*
Timing of cigarette use initiation	19-20yo	2.51 (.52, 12.22)	.25	.79 (.54, 1.16)	.23	1.24 (.96, 1.61)	.10
	16-18yo	1.02 (.26, 4.03)	.98	.98 (.76, 1.28)	.90	1.08 (.86, 1.36)	.50
	< 15yo	**4.24 (1.35, 13.28)**	**.01**	**1.31 (1.04, 1.64)**	**.02**	.89 (.73, 1.09)	.27
Timing of alcohol use initiation	19-20yo	1.82 (.38, 8.80)	.45	.93 (.67, 1.30)	.68	.90 (.70, 1.15)	.39
	16-18yo	**6.00 (1.08, 33.35)**	**.04**	1.14 (.81, 1.61)	.46	.78 (.58, 1.04)	.09
	< 15yo	3.60 (.82, 15.78)	.09	1.06 (.78, 1.42)	.72	.94 (.74, 1.20)	.62
Timing of marijuana use initiation	19-20yo	2.10 (.52, 8.50)	.30	.88 (.63, 1.24)	.47	.92 (.71, 1.20)	.54
	16-18yo	.88 (.24, 3.25)	.85	1.09 (.85, 1.40)	.50	1.13 (.92, 1.38)	.25
	< 15yo	**4.40 (1.09, 17.75)**	**.04**	1.24 (.95, 1.63)	.12	.86 (.68, 1.08)	.19

(Reference Group = Initiated at age 19–20)							
		Physical Victimization		Verbal Victimization		Relational Victimization	
		*RRR (95% CI)*	*p*	*RRR (95% CI)*	*p*	*RRR (95% CI)*	*p*
Timing of cigarette use initiation	No use by 21yo	.40 (.08, 1.94)	.25	1.26 (.86, 1.86)	.23	.80 (.62, 1.04)	.10
	16-18yo	.40 (.06, 2.69)	.35	1.24 (.81, 1.92)	.32	.87 (.63, 1.20)	.39
	< 15yo	1.69 (.30, 9.63)	.55	**1.65** **(1.09, 2.51)**	**.02**	**.72 (.53, .97)**	**.03**
Timing of alcohol use initiation	No use by 21yo	.55 (.11, 2.65)	.45	1.07 (.77, 1.50)	.68	1.11 (.87, 1.42)	.39
	16-18yo	3.29 (.53, 20.42)	.20	1.22 (.83, 1.79)	.31	.87 (.63, 1.19)	.38
	< 15yo	1.98 (.39, 10.02)	.41	1.13 (.80, 1.59)	.48	1.05 (.79, 1.38)	.75
Timing of marijuana use initiation	No use by 21yo	.48 (.12, 1.93)	.30	1.13 (.81, 1.58)	.47	1.09 (.83, 1.41)	.54
	16-18yo	.42 (.08, 2.24)	.31	1.24 (.84, 1.81)	.27	1.22 (.91, 1.65)	.19
	< 15yo	2.10 (.38, 11.72)	.40	1.41 (.95, 2.08)	.09	.93 (.68, 1.28)	.66

(Reference Group = Initiated at age 16–18)							
		Physical Victimization		Verbal Victimization		Relational Victimization	
		*RRR (95% CI)*	*p*	*RRR (95% CI)*	*p*	*RRR (95% CI)*	*p*
Timing of cigarette use initiation	No use by 21yo	.98 (.25, 3.90)	.98	1.02 (.78, 1.32)	.90	.92 (.73, 1.17)	.50
	19-20yo	2.47 (.37, 16.44)	.35	.80 (.52, 1.24)	.32	1.15 (.84, 1.58)	.39
	< 15yo	4.17 (.92, 19.01)	.07	1.33 (.99, 1.79)	.06	.83 (.63, 1.08)	.16
Timing of alcohol use initiation	No use by 21yo	**.17 (.03, .93)**	**.04**	.88 (.62, 1.24)	.46	1.29 (.96, 1.73)	.09
	19-20yo	.30 (.05, 1.89)	.20	.82 (.56, 1.20)	.31	1.16 (.84, 1.59)	.38
	< 15yo	.60 (.11, 3.35)	.56	.93 (.65, 1.31)	.67	1.21 (.91, 1.61)	.20
Timing of marijuana use initiation	No use by 21yo	1.13 (.31, 4.18)	.85	.92 (.71, 1.18)	.50	.89 (.72, 1.09)	.25
	19-20yo	2.38 (.45, 12.65)	.31	.81 (.55, 1.18)	.28	.82 (.60, 1.10)	.19
	< 15yo	**4.98 (1.00, 24.73)**	**.05**	1.14 (.83, 1.57)	.43	**.76 (.58, 1.00)**	**.05**

Note: For timing of substance use initiation variables, Level 1 = Never endorsed by the final assessment: if the child/adolescent never reported ever use in any wave; Level 2 = Late initiation: if the child/adolescent reported the first ever use at age 19–20 (wave 5); Level 3 = Mid initiation: if the child/adolescent reported the first ever use at age 16–18 (wave 4); Level 4 = Early initiation: if the child/adolescent reported the first ever use at or before age 15 (wave 1–3). The number of observations is 644 for cigarette initiation models, 413 for alcohol initiation models, and 589 for marijuana initiation models

### Biometric Analyses

#### Cross-Twin Within-Trait and Cross-Trait Correlations

The phenotypic correlation matrix (treating timing of substance use initiation as continuous) is demonstrated in Table 7, twin correlations are shown in Table 8, the cross-twin cross-trait correlations are shown in Table 9, and the within-twin concordance for the three substance use initiation variables can be found in Table 10. Results suggested that all three peer victimization variables were moderately correlated (*r*’s = 0.41–0.56), which supports further examining them as three separate predictors. All three substance initiation variables were very highly correlated (*r*’s = 0.74–0.80), which is consistent with previous findings revealing high rate of co-use of multiple types of substances in drug users (Roche et al., 2019).

**Table 7:** Phenotypic Correlation Matrix for Peer Victimization and Substance Initiation Variables

Variable	1	2	3	4	5	6
1 Physical Victimization	1					
2 Verbal Victimization	.56***	1				
3 Relational Victimization	.41***	.56***	1			
4 Timing of Cigarette Use Initiation	.15***	.16***	.05	1		
5 Timing of Alcohol Use Initiation	.09	.09	.01	.74***	1	
6 Timing of Marijuana Use Initiation	.09*	.11**	.04	.80***	.78***	1

Note: .08 < ^+^p < .05, *p < .05, **p < .01, ***p < .001

**Table 8:** Twin Correlation Matrix for Peer Victimization and Timing of Substance Use Initiation Variables

	1 Physical Victimization	2 Verbal Victimization	3 Relational Victimization	4 Timing of Cigarette Use Initiation	5 Timing of Alcohol Use Initiation	6 Timing of Marijuana Use Initiation
MZ vs. DZ						
MZ	.33	.41	.23	.77	.88	.85
DZ	.24	.27	.26	.56	.67	.58
MZF vs. DZF						
MZF	.33	.35	.15	.74	.86	.83
DZF	.11	.24	.09	.59	.88	.76
MZM vs. DZM						
MZM	.33	.48	.28	.81	.90	.87
DZM	.27	.23	.23	.40	.67	.55
DZOS						
DZOS	.26	.27	.27	.64	.54	.49

MZF = MZ female twin pairs, DZF = DZ female twin pairs, MZM = MZ male twin pairs, DZM = DZ male twin pairs, DZOS = DZ opposite-sex pairs

**Table 9:** Cross-twin Cross-trait Correlation Matrix of Main Variables for MZ and DZ Twin Pairs (DZ Correlations Below the Diagonal)

	T1 VV	T1 PV	T1 RV	T1 ToC	T1 ToA	T1 ToM	T2 VV	T2 PV	T2 RV	T2 ToC	T2 ToA	T2 ToM
T1 VV	1.00	.53	.46	.27	.15	.21	.41	.28	.23	.23	.10	.21
T1 PV	.62	1.00	.29	.26	.18	.18	.31	.33	.29	.22	.29	.22
T1 RV	.62	.44	1.00	.07	.06	.02	.24	.27	.22	.01	− .00	− .01
T1 ToC	.10	.02	.06	1.00	.71	.82	.26	.21	.10	.77	.72	.73
T1 ToA	− .01	− .13	− .10	.69	1.00	.75	.32	.32	.16	.71	.88	.71
T1 ToM	.07	.10	.06	.77	.69	1.00	.22	.17	.09	.75	.75	.84
T2 VV	.25	.18	.20	− .07	− .26	− .14	1.00	.60	.58	.29	.22	.25
T2 PV	.20	.21	.24	.01	− .04	.02	.53	1.00	.46	.21	.23	.15
T2 RV	.21	.19	.23	− .06	− .24	− .04	.55	.44	1.00	.09	.08	.13
T2 ToC	.08	.03	− .03	.56	.60	.51	.02	.17	− .10	1.00	.81	.81
T2 ToA	.15	.07	.02	.49	.66	.55	.03	.12	.01	.74	1.00	.85
T2 ToM	.08	− .02	− .06	.47	.58	.58	− .04	.07	− .02	.79	.83	1.00

Note: T1 = Twin 1; T2 = Twin 2; VV = Verbal Victimization; PV = Physical Victimization; RV = Relational Victimization; ToC = Timing of cigarette use initiation; ToA = Timing of alcohol use initiation; ToM = Timing of marijuana use initiation

**Table 10:** Within-twin Concordance for Timing of Substance Use Initiation Variables

Timing of cigarette use initiation					
		MZ Twin 1			
		Level 1	Level 2	Level 3	Level 4
MZ Twin 2	Level 1	105 (60.69%)	5 (2.89%)	4 (2.31%)	2 (1.16%)
	Level 2	5 (2.89%)	3 (1.73%)	2 (1.16%)	1 (.58%)
	Level 3	2 (1.16%)	1 (.58%)	13 (7.51%)	5 (2.89%)
	Level 4	3 (1.73%)	3 (1.73%)	2 (1.16%)	17 (9.83%)
		DZ Twin 1			
		Level 1	Level 2	Level 3	Level 4
DZ Twin 2	Level 1	97 (42.73%)	6 (2.64%)	10 (4.41%)	11 (4.85%)
	Level 2	12 (5.29%)	6 (2.64%)	5 (2.20%)	1 (.44%)
	Level 3	10 (4.41%)	5 (2.20%)	11 (4.85%)	12 (5.29%)
	Level 4	8 (3.52%)	1 (.44%)	5 (2.20%)	27 (11.89%)

Timing of alcohol use initiation					
		MZ Twin 1			
		Level 1	Level 2	Level 3	Level 4
MZ Twin 2	Level 1	44 (43.56%)	7 (6.93%)	1 (.99%)	1 (.99%)
	Level 2	4 (3.96%)	7 (6.93%)	4 (3.96%)	1 (.99%)
	Level 3	0 (.00%)	2 (1.98%)	6 (5.94%)	2 (1.98%)
	Level 4	0 (.00%)	0 (.00%)	2 (1.98%)	20 (19.80%)
DZ Twin 1					
		Level 1	Level 2	Level 3	Level 4
DZ Twin 2	Level 1	26 (27.96%)	5 (5.38%)	3 (3.23%)	2 (2.15%)
	Level 2	3 (3.23%)	8 (8.60%)	4 (4.30%)	3 (3.23%)
	Level 3	2 (2.15%)	2 (2.15%)	7 (7.53%)	3 (3.23%)
	Level 4	3 (3.23%)	0 (.00%)	3 (3.23%)	19 (20.43%)

Timing of marijuana use initiation					
		MZ Twin 1			
		Level 1	Level 2	Level 3	Level 4
MZ Twin 2	Level 1	90 (58.06%)	5 (3.23%)	3 (1.94%)	1 (.65%)
	Level 2	3 (1.94%)	8 (5.16%)	2 (1.29%)	0 (.00%)
	Level 3	3 (1.94%)	4 (2.58%)	19 (12.26%)	1 (.65%)
	Level 4	0 (.00%)	1 (.65%)	3 (1.94%)	12 (7.74%)
		DZ Twin 1			
		Level 1	Level 2	Level 3	Level 4
DZ Twin 2	Level 1	72 (36.55%)	9 (4.57%)	10 (5.08%)	8 (4.06%)
	Level 2	10 (5.08%)	13 (6.60%)	3 (1.52%)	3 (1.52%)
	Level 3	9 (4.57%)	4 (2.03%)	20 (10.15%)	4 (2.03%)
	Level 4	2 (1.02%)	3 (1.52%)	6 (3.05%)	21 (10.66%)

Note: For timing of substance use initiation variables, Level 1 = Never endorsed by the final assessment: if the child/adolescent never reported ever use in any wave; Level 2 = Late initiation: if the child/adolescent reported the first ever use at age 19–20 (wave 5); Level 3 = Mid initiation: if the child/adolescent reported the first ever use at age 16–18 (wave 4); Level 4 = Early initiation: if the child/adolescent reported the first ever use at or before age 15 (wave 1–3)

However, the correlations between peer victimization and timing of substance use initiation variables were quite small in magnitude (*r’*s = 0.01–0.16). The only significant correlations between peer victimization and timing of substance use initiation variables were: (1) higher physical victimization was correlated with earlier initiation of cigarette (*r* = 0.15, *p* < 0.001) and marijuana (*r* = 0.09, *p* = 0.03); and (2) higher verbal victimization was correlated with earlier initiation of cigarette (*r* = 0.16, *p* < 0.001) and marijuana use (*r* = 0.11, *p* = 0.01). Given that the proposed multivariate analyses (i.e., Cholesky Decomposition) require bivariate correlations among variables to be higher than 0.20, we were unable to proceed with multivariate modeling (Keskitalo et al. 2007; Tabachnick & Fidell 2018).

When running univariate genetic models, the means and variances were unable to be constrained to the same across zygosity types for relational victimization, and the estimators were therefore allowed to be free for MZ and DZ twins in subsequent models. The other five main variables passed the equal mean and variance assumption check. In sex limitation models, alcohol and marijuana initiation variables suggested quantitative sex differences. Thus, variance components were fitted separately for males and females for these two variables Table 11.

**Table 11:** Univariate Twin Model Parameter Estimates and Fit Statistics

Model	A	C	E	*AIC*	*BIC*	*-2LL*	*p*
Verbal Victimization							
ACE	.33 [.04, .51]	.09 [.00, .31]	.58 [.49, .69]	3558.20	3575.61	3533.79	–
** AE**	**.43 [.34, .51]**	**–**	**.57 [.49, .66]**	**3556.72**	**3572.71**	**3534.37**	**.45**
CE	–	.32 [.25, .40]	.68 [.60, .75]	3561.10	3577.09	3538.76	.03
E	–	–	1.00 [1.00, 1.00]	3618.82	3633.38	3598.53	< .001
Physical Victimization							
ACE	.21 [.00, .42]	.11 [.00, .32]	.68 [.58, .79]	3602.97	3620.38	3578.56	–
** AE**	**.34 [.24, .43]**	**-**	**.66 [.57, .76]**	**3601.79**	**3617.78**	**3579.44**	**.35**
** CE**	**–**	**.26 [.18, .34]**	**.74 [.66, .82]**	**3602.72**	**3618.71**	**3580.38**	**.18**
E	**–**	**–**	1.00 [1.00, 1.00]	3640.70	3655.26	3620.41	< .001
Relational Victimization							
ACE	.00 [.00, .27]	.22 [.01, .30]	.78 [.68, .86]	3870.15	3887.56	3845.74	**–**
AE	.26 [.16, .35]	**–**	.74 [.65, .84]	3872.16	3888.15	3849.81	.04
** CE**	**–**	**.22 [.14, .30]**	**.78 [.70, .86]**	**3868.09**	**3884.08**	**3845.74**	**1.00**
E	**–**	**–**	1.00 [1.00, 1.00]	3894.26	3908.83	3873.97	< .001
Timing of cigarette use initiation							
** ACE**	**.48 [.31, .68]**	**.31 [.12, .46]**	**.21 [.17, .26]**	**2776.05**	**2793.46**	**2751.64**	**–**
AE	.80 [.75, .84]	**–**	.20 [.16, .25]	2783.72	2799.71	2761.37	.00
CE	**–**	.65 [.59, .70]	.35 [.30, .41]	2802.08	2818.08	2779.74	< .001
E	**–**	**–**	1.00 [1.00, 1.00]	3013.58	3028.14	2993.29	< .001
Timing of alcohol use initiation							
ACE_M,F_	A_M_ = .54 [.23, .92]	C_M_ = .36 [.00, .67]	E_M_ = .10 [.01, .16]	1308.77	1325.77	1277.92	**–**
	A_F_ = .00 [.00, .19]	C_F_ = .86 [.67, .90]	E_F_ = .14 [.01, .20]				
AE_M,F_	A_M_ = .90 [.84, .93]	**–**	E_M_ = .10 [.07, .16]	1327.17	1342.00	1300.53	< .001
	A_F_ = .86 [.78, .90]	**–**	E_F_ = .14 [.10, .22]				
CE_M,F_	**–**	C_M_ = .81 [.73, .87]	E_M_ = .19 [.13, .27]	1320.39	1335.22	1293.75	< .001
	**–**	C_F_ = .86 [.80, .90]	E_F_ = .14 [.10, .20]				
** AE**_**M**_**, CE**_**F**_	**A**_**M**_** = .90 [.84, .93]**	**–**	**E**_**M**_** = .10 [.07, .16]**	**1306.46**	**1321.29**	**1279.82**	**.39**
	**–**	**C**_**F**_** = .86 [.80, .90]**	**E**_**F**_** = .14 [.10, .20]**				
E_M,F_	**–**	**–**	E_M_ = 1.00 [1.00, 1.00]	1502.59	1515.22	1480.13	< .001
	**–**	**–**	E_F_ = 1.00 [1.00, 1.00]				
Timing of marijuana use initiation							
ACE_M,F_	A_M_ = .66 [.37, .92]	C_M_ = .23 [.00, .52]	E_M_ = .11 [.01, .16]	1677.39	1699.04	1646.76	**–**
	A_F_ = .15 [.00, .39]	C_F_ = .67 [.44, .82]	E_F_ = .18 [.13, .26]				
AE_M,F_	A_M_ = .89 [.84, .92]	**–**	E_M_ = .11 [.08, .16]	1693.39	1712.22	1666.92	< .001
	A_F_ = .82 [.76, .87]	**–**	E_F_ = .18 [.13, .24]				
CE_M,F_	**–**	C_M_ = .76 [.68, .82]	E_M_ = .24 [.18, .32]	1704.58	1723.41	1678.11	< .001
	**–**	C_F_ = .79 [.72, .84]	E_F_ = .21 [.16, .28]				
** AE**_**M**_**, CE**_**F**_	**A**_**M**_** = .89 [.84, .92]**	**–**	**E**_**M**_** = .11 [.08, .16]**	**1676.64**	**1695.47**	**1650.17**	**.18**
	**–**	**C**_**F**_** = .79 [.72, .84]**	**E**_**F**_** = .21 [.16, .28]**				
E_M,F_	**–**	**–**	E_M_ = 1.00 [1.00, 1.00]	1938.62	1954.61	1916.27	< .001
	**–**	**–**	E_F_ = 1.00 [1.00, 1.00]				

Note: AE and CE sub-model fitted equally for physical victimization, thus variance components for both sub-models were reported. For timing of alcohol and marijuana use initiation, variance components were estimated separately for males and females, with M = male, F = female, in subscripts of model names. Models in bold texts are best fitting models

#### Univariate Model Results

According to the comparison between MZ and DZ phenotypic correlations for each main variable, ACE models were chosen for all main variables. Subsequently, A, C, and both A and C together were omitted from the full model in sequence, to detect the best fitting and most parsimonious full or nested model for each variable, respectively. The twins’ age and dummy-coded race/ethnicity entered each model as covariates. Results of variance components are shown in Table 11.

The best fitting model of verbal victimization is the AE model (V_A_ = 0.43 [0.34, 0.51], V_E_ = 0.57 [0.49, 0.66], *-2LL* = 3534.37, *p* = 0.45). Either A or C could be dropped from the saturated model of physical victimization, which implied ambiguous familial influences (AE model: V_A_ = 0.34 [0.24, 0.43], V_E_ = 0.66 [0.57, 0.76], *-2LL* = 3579.44, *p* = 0.35; CE model: V_C_ = 0.26 [0.18, 0.34], V_E_ = 0.74 [0.66, 0.82], *-2LL* = 3580.38, *p* = 0.18). For relational victimization, the CE model was the best fitting, parsimonious model (V_C_ = 0.22 [0.14, 0.30], V_E_ = 0.78 [0.70, 0.86], *-2LL* = 3845.74, *p* = 1.00). Neither A nor C could be dropped for timing of cigarette use initiation model, which made the ACE model the best fitting model (V_A_ = 0.48 [0.31, 0.68], V_C_ = 0.31 [0.12, 0.46], V_E_ = 0.21 [0.17, 0.26], *-2LL* = 2751.64). For timing of alcohol use initiation model, the C could be dropped for males, while the A could be dropped for females (V_AM_ = 0.90 [0.84, 0.93], V_EM_ = 0.10 [0.07, 0.16]; V_CF_ = 0.86 [0.80, 0.90], V_EF_ = 0.14 [0.10, 0.20]; *-2LL* = 1279.82, *p* = 0.39). Similarly, for timing of marijuana use initiation, AE for male and CE for female was the best fitting and most parsimonious model, while the genetic contributions to males and shared environmental contributions to females were substantial (V_AM_ = 0.89 [0.84, 0.92], V_EM_ = 0.11 [0.08, 0.16]; V_CF_ = 0.79 [0.72, 0.84], V_EF_ = 0.21 [0.16, 0.28]; *-2LL* = 1650.17, *p* = 0.18).

#### Addressing Aim 2: Multivariate Results

Given that the cross-trait correlations between initiation and victimization were very small in magnitude (*r*’s = 0.01 to 0.16, see Table 7), we were unable to run multivariate twin analyses.

### Sensitivity Analysis

Sensitivity analyses were conducted by rerunning all analyses in the subsample with complete data (237 twin pairs), in which both co-twins have at least one peer victimization variable and at least one substance initiation variable. This data cleaning method was selected given that (1) 176 twin pairs dropped by Wave 3, which may bias the inference of timing of substance initiation; and (2) this data cleaning criterion has been used in multiple articles using RFAB data (e.g., Bertoldi et al., 2023). The results are shown in *Supplementary Materials*, Tables S3.1–S3.11. Mixed-effect models in sensitivity analysis yielded less findings than main analysis, such that physical victimization did not show sex differences, and no racial/ethnic differences were found for any main variables. This might be due to the lack of statistical power in the sensitivity analysis sample.

In multinomial logistic regressions, findings were fairly consistent with main analyses, although in some cases findings were stronger (and nominally significant) in the main analysis but not the sensitivity analysis, and in other cases findings were stronger (and nominally significant) in the sensitivity analysis but not the main analysis. Again, no findings survived Bonferroni, Holm-Bonferroni, or Benjamini–Hochberg correction. As in the main analysis, given the low correlations between peer victimization and substance use initiation variables (*r’*s = − 0.01–0.17), the multivariate twin analyses were unable to be conducted.

In univariate twin analyses, sensitivity analysis showed different findings for relational victimization and all three substance use initiation variables. Ambiguous familial influences were suggested in the univariate models of relational victimization, revealing either AE (V_A_ = 0.20 [0.04, 0.34], V_E_ = 0.80 [0.66, 0.96], *-2LL* = 1586.90, *p* = 0.93) or CE (V_C_ = 0.15 [0.02, 0.27], V_E_ = 0.85 [0.73, 0.98], *-2LL* = 1587.37, *p* = 0.49) model as the best fitting model. For both cigarette (V_A_ = 0.80 [0.73, 0.85], V_E_ = 0.20 [0.15, 0.27], *-2LL* = 1255.01, *p* = 0.24) and alcohol initiation (V_A_ = 0.85 [0.78, 0.90], V_E_ = 0.15 [0.10, 0.22], *− 2LL* = 812.82, *p* = 0.10), the best fitting univariate model was the AE model. The best fit for the univariate model regarding marijuana initiation was provided by the full ACE model (V_A_ = 0.43 [0.18, 0.71], V_C_ = 0.36 [0.09, 0.57], V_E_ = 0.22 [0.16, 0.30], *-2LL* = 1087.82). There were no sex differences in univariate models.

## Discussion

The present study aimed to investigate the associations between childhood victimization experience and timing of substance use initiation (Aim 1), and genetic and environmental contributions to these associations (Aim 2). After controlling for adolescent sex, age, and race/ethnicity, there were few significant associations (9/54; 16.7%) and none survived corrections for multiple testing; further, the overall patterns of findings were not systematic. In addition, associations treating timing of initiation variables as continuous indicators of severity yielded very small associations that precluded bivariate decomposition of associations (Aim 2). Thus, our hypotheses were generally not supported, and call into question the strength of associations between middle childhood bullying and timing of substance use initiation measured prospectively across adolescence.

More consistent with past literature, univariate decompositions in the main analyses with the full sample revealed that verbal victimization was influenced by A and E, relational victimization was explained by C and E, whereas physical victimization included ambiguous familial influences. Sex differences were revealed for timing of alcohol and marijuana use initiation, with genetic factors playing an important role for male’s alcohol and marijuana use initiation, while shared environmental factors were more pronounced for females.

### The Link Between Peer Victimization and Substance Use

Both the main and sensitivity analyses indicated that the experience of physical and verbal victimization in middle childhood could increase the risk of initiating smoking by age 15, which is consistent with previous evidence (Earnshaw et al. 2017). Children who are victimized by their peers, either physically or verbally, may experience negative affect and encounter academic difficulties, and therefore resort to smoking or vaping as a way to release stress (Vannucci et al. 2021). However, it is unclear whether these results are interpretable, given that these effects were very small and very sparse and did not survive adjustment for multiple testing.

Higher levels of relational victimization, however, increased the possibility of initiating cigarette smoking at specifically the age 19–20 assessment, rather than at earlier (< 15y) ages, in both main and sensitivity analyses. This is inconsistent with prior work showing that relational victimization increases the risk of early cigarette use (Sullivan et al. 2006). Speculatively, this assessment timing is the first assessment after the age of legal cigarette use and may indicate that, while relational bullying victims are at increased risk of cigarette use, they may wait to initiate until it is no longer illegal or until they gain sufficient independence in college (Johnson et al. 2019; Rice et al. 1995). In addition, those bullied children may initiate smoking e-cigarettes first, given recent findings suggesting more associations between relational victimization and using e-cigarette products (e.g., e-cigarette, vape pen, or e-hookah) (Azagba et al. 2020), which was not captured by the RFAB.

Relational victimization showed a surprisingly protective effect on adolescents, as individuals faced more relational exclusion from peers tended to delay their timing of alcohol use initiation to later than age 19–20, if at all. If real, this finding could reflect that children choose (or have) to stay away from mainstream peer groups (Casper et al. 2020), and further get less involved with the social occasions that encourage alcohol use. However, this effect is possibly a statistical artifact driven by the large proportion of adolescents who never endorsed alcohol use (see Figure S2), because this result only showed in sensitivity analysis.

The finding of most comparisons did not pass any correction for multiple testing may be attributed to the measure that we used. Our study used a very conservative way to infer the timing of substance initiation, with most cases with partial attrition being eliminated during data cleaning. For example, a participant who reported “Missing, Missing, No, Missing, Yes” on ever use across the 5 waves of data collection was excluded because we could not determine whether they initiated before Wave 4 (age 16–18) vs. Wave 5 (age 19–20). This contributed to the small sample sizes in some timing of substance initiation categories.

Another explanation of our null findings may be due to using a twin sample. Abundant research findings suggest that support from family members and support from friends can attenuate the association between peer victimization and early substance use risks (Miller et al. 2014; Vannucci et al. 2021). Co-twins are undoubtedly at comparable ages to adolescent’s peers, and thus relationships with co-twins may act in double support roles as both family relationship and peer relationship (Stocker & Dunn 1990), and promote individual’s socially adaptive behaviors (Pulkkinen et al. 2003). Therefore, our twin sample may naturally imply the moderating role of having same-age siblings by providing support and/or monitoring, which attenuates the associations between peer victimization and timing of substance initiation that could be more profound in singletons.

### Genetic and Environmental Contributions

Although multivariate biometric models were not feasible due to low phenotypic correlations across victimization variables and timing of substance use initiation variables, we performed univariate decomposition for each single variable. Environmental factors that are not shared by co-twins were found to account for more than half of the variance of all three forms of peer victimization, in both main analyses and sensitivity analysis. This is contrary to the findings of Veldkamp et al. (2019), who reported that A explained a substantial proportion of the variance for bullying victimization, while E only accounted for ~ 15–20% of the variability. Veldkamp’s study, however, focused on a demographically different sample (i.e., Netherland children aged 6–13) from ours, which may explain the distinct findings.

The remaining proportion of the variance in verbal victimization all went to A, supporting the consensus from prior studies that genetic influences play an important role in the occurrence of verbal victimization among twins (Johansson et al. 2022; Veldkamp et al. 2019). Given that peer victimization is an exposure to peer contexts rather than a straightforward behavior, the heritability may be attributed to other heritable phenotypes (e.g., social reticence, depressive symptoms, anxiety) that may increase the susceptibility to verbal victimization (Guimond et al. 2018; Mlawer et al. 2019). Additionally, the susceptibility to verbal victimization may be elevated through active gene-environment correlation (i.e., active *r*GE). Specifically, children tend to select into certain peer groups according to their phenotypes (e.g., personality) influenced by genetic predispositions, which can be detrimental to individual’s developmental outcomes (TenEyck & Barnes 2015).

In the main analysis, the CE model provided the best fit for relational victimization, while physical victimization revealed ambiguous familial influences. In sensitivity analysis, however, both forms of victimization suggested ambiguous familial influences. This underscores the importance of using larger sample sizes and leveraging analytical methods with better power. Moreover, the persistent ambiguous familial influences necessitate larger samples with more sibling types, or measures capturing broader aspects of victimization, to discern the unique contributions of A versus C.

In the main analysis, cigarette use initiation revealed substantial genetic influences, which is highly consistent with previous evidence indicating that genetic contributions to substance initiation remain moderate to high across multiple age groups (Richmond-Rakerd et al. 2016; Zellers et al. 2022). Sex differences, however, were suggested for alcohol and marijuana use initiation, with genetic factors could be omitted for females, and shared environmental factors could be dropped for males. These observed sex differences align with previous twin studies of alcohol and marijuana use initiation, which suggest that genetic factors have more important influences on males, while shared environmental factors exert more profound impacts in females (Poelen et al. 2008; Verweij et al. 2010). Multiple studies examining interaction effects of sex and alcohol use polygenic risk scores (PRS) also revealed that PRSs showed stronger predicting roles for alcohol use outcomes among males (Chang et al. 2019; Kranzler et al. 2023), while no moderating role of sex was found for cigarette initiation (Chang et al. 2019). The observed sex differences in marijuana use initiation are novel and warrant careful examination in future PRS studies, to reveal potential mechanism underlying these differences. The sex differences, however, were not detected in the sensitivity analysis including 237 twin pairs, which emphasizes the importance to leverage samples with larger sample sizes and to conduct analysis with methods of higher statistical power.

## Strengths, Limitations, and Conclusions

The present study has several positive features. First, the use of ever use of substances to infer timing of substance initiation in a longitudinal design helped to minimize the recall bias. Second, we examined the variance components and the impacts on timing of substance use initiation separately for subtypes of peer victimization, corroborating previous evidence pointing out differentiated contributions of A, C, and E for subtypes (Veldkamp et al. 2019). Third, timing of substance use initiation was investigated carefully based on four categories—early, mid, and late initiation, and never endorsed by the final assessment – according to the distribution of initiation rate across age groups. This categorization helped capture the potential effects of age-related social transitions (e.g., transitioning from high school to college, getting more independence after freshman year) on initiating alcohol, cigarette, and marijuana use at specific timepoints (Rice et al. 1995).

This study also bears several limitations. First, all main variables were measured via child and adolescent self-report, which may be possibly biased due to social desirability and thus, may exaggerate the correlation across constructs, and may be less reliable at the earlier waves of data collection (Jimerson et al. 2012). This shared method variance is likely to inflate associations, but our associations were very sparse and small, so any bias is likely not meaningful. Future studies should consider incorporating reports from multiple informants, such as peers (Rodkin & Berger 2008). Second, the lacking in children’s bullying perpetration data left out the potential differences between children with victim and bully-victim profiles. Children with different bullying profiles, however, show different developmental trajectories in later life, with bully-victims being particularly susceptible to underage substance use and substance use disorders (Weiss et al. 2011). Different profiles, on the other hand, might reveal differentiated contributions of A, C, and E (Ball et al. 2008; Guy et al. 2019). Future studies should include bullying perpetration data to either extract multiple profiles of bullying experiences or control in phenotypic regression and biometric analyses. Third, the present study only included monozygotic twins, same-sex dizygotic twins, and opposite-sex dizygotic twins. The lack of more types of siblings (e.g., full siblings, half-siblings) prevented us to further disentangling the sources of ambiguous familial influences. Future studies may consider leveraging larger sized samples with multiple sibling types. Fourth, the sample sizes of some timing of substance initiation levels in the multinomial logistic regression were very low, which might be due to the very conservative way of inferring timing categories that we used. Sixth, the current study did not incorporate the temperament and personality factors (e.g., social deficits, impulsivity, shyness) that may underlie peer victimization and substance initiation and explain the detected genetic and environmental influences on our main constructs (Ball et al. 2008; Mynard & Joseph 1997). Future studies can consider including those precursors and conduct multivariate decompositions to examine this question. Seventh, the substance use initiation time was inferred from participants binary (yes/no) reports at each wave of data collection. This categorical measurement reduced variance in the variables and may impact statistical power. Future studies are encouraged to collect continuous data on substance use initiation to improve precision.

Despite these limitations, the present study offers new insights into the link between childhood physical, verbal, and relational victimization and timing of substance use initiation in various stages. After controlling for adolescent’s age, sex, and race/ethnicity, no comparison in the multinomial logistic regression passed the Bonferroni-corrected threshold in either the main or sensitivity analysis. This raised the possibility that the association between peer victimization and substance use is not as strong as previous evidence shows. From a genetically informed perspective, males and females yielded different contributions of A and C to timing of alcohol and marijuana use initiation. Several differences are suggested between the main and sensitivity analyses, which underscore the importance to utilize analytical designs with better power.

## Supplementary Information

Below is the link to the electronic supplementary material.

Supplementary file1 (DOCX 709 kb)

## Data Availability

The data that support the findings of this study are available from study of Risk Factors for Antisocial Behavior (RFAB), but restrictions apply to the availability of these data. The data are available from the authors upon request and with the permission of the team of RFAB.

## References

[CR1] Azagba S, Mensah NA, Shan L, Latham K (2020) Bullying victimization and e-cigarette use among middle and high school students. J Sch Health 90(7):545–553. 10.1111/josh.12902 32406087 10.1111/josh.12902

[CR2] Baker LA, Barton M, Lozano DI, Raine A, Fowler JH (2006) The Southern California twin register at the university of Southern California: II. Twin Res Hum Genet 9(6):933–940. 10.1375/twin.9.6.933 17254433 10.1375/183242706779462912 PMC1913188

[CR3] Baker LA, Tuvblad C, Wang P, Gomez K, Bezdjian S, Niv S, Raine A (2013) The Southern California Twin register at the university of Southern California: III. Twin Res Hum Genet 16(1):336–343. 10.1017/thg.2012.127 23394193 10.1017/thg.2012.127 PMC3674229

[CR4] Ball HA, Arseneault L, Taylor A, Maughan B, Caspi A, Moffitt TE (2008) Genetic and environmental influences on victims, bullies and bully-victims in childhood. J Child Psychol Psychiatry 49(1):104–112. 10.1111/j.1469-7610.2007.01821.x 18181884 10.1111/j.1469-7610.2007.01821.x

[CR97] Bertoldi BM, Tuvblad C, Joyner KJ, Ganley C, Raine A, Baker L, Latvala A, Oskarsson S, Patrick CJ (2023) Role of triarchic traits in relations of early resting heart rate with antisocial behavior and broad psychopathology dimensions in later life. Clin Psychol Sci 11(1):90–105. 10.1177/21677026221081880

[CR5] Biswas T, Scott JG, Munir K, Thomas HJ, Huda MM, Hasan MdM, David de Vries T, Baxter J, Mamun AA (2020) Global variation in the prevalence of bullying victimisation amongst adolescents: role of peer and parental supports. EClinicalMedicine 20:100276. 10.1016/j.eclinm.2020.100276 32300737 10.1016/j.eclinm.2020.100276 PMC7152826

[CR6] Boulton MJ, Trueman M, Flemington I (2002) Associations between secondary school pupils’ definitions of bullying, attitudes towards bullying, and tendencies to engage in bullying: age and sex differences. Educ Stud 28(4):353–370. 10.1080/0305569022000042390

[CR7] Brendgen M, Boivin M, Vitaro F, Girard A, Dionne G, Pérusse D (2008) Gene–environment interaction between peer victimization and child aggression. Dev Psychopathol 20(2):455–471. 10.1017/S0954579408000229 18423089 10.1017/S0954579408000229

[CR8] Cañas E, Estevez E, Estevez JF (2022) Sociometric status in bullying perpetrators: a systematic review. Front Commun. 10.3389/fcomm.2022.841424

[CR9] Carbone-Lopez K, Esbensen F-A, Brick BT (2010) Correlates and consequences of peer victimization: gender differences in direct and indirect forms of bullying. Youth Violence Juvenile Justice 8(4):332–350. 10.1177/1541204010362954

[CR10] Casper DM, Card NA (2017) Overt and relational victimization: a meta-analytic review of their overlap and associations with social–psychological adjustment. Child Dev 88(2):466–483. 10.1111/cdev.12621 27709610 10.1111/cdev.12621

[CR11] Casper DM, Card NA, Barlow C (2020) Relational aggression and victimization during adolescence: a meta-analytic review of unique associations with popularity, peer acceptance, rejection, and friendship characteristics. J Adolesc 80:41–52. 10.1016/j.adolescence.2019.12.012 32062169 10.1016/j.adolescence.2019.12.012

[CR12] Chang Z, Lichtenstein P, Larsson H (2012) The effects of childhood ADHD symptoms on early-onset substance use: a Swedish twin study. J Abnorm Child Psychol 40(3):425–435. 10.1007/s10802-011-9575-6 21947618 10.1007/s10802-011-9575-6

[CR13] Chang L-H, Whitfield JB, Liu M, Medland SE, Hickie IB, Martin NG, Verhulst B, Heath AC, Madden PA, Statham DJ, Gillespie NA (2019) Associations between polygenic risk for tobacco and alcohol use and liability to tobacco and alcohol use, and psychiatric disorders in an independent sample of 13,999 Australian adults. Drug Alcohol Depend 205:107704. 10.1016/j.drugalcdep.2019.107704 31731259 10.1016/j.drugalcdep.2019.107704

[CR14] Connolly EJ (2017) Sex differences in childhood bullying victimization and trajectories of substance use from adolescence to adulthood. J Drug Issues 47(1):25–49. 10.1177/0022042616678605

[CR15] Crick NR, Grotpeter JK (1995) Relational aggression, gender, and social-psychological adjustment. Child Dev 66(3):710–722. 10.1111/j.1467-8624.1995.tb00900.x 7789197 10.1111/j.1467-8624.1995.tb00900.x

[CR16] Davis JP, Dumas TM, Merrin GJ, Espelage DL, Tan K, Madden D, Hong JS (2018) Examining the pathways between bully victimization, depression, academic achievement, and problematic drinking in adolescence. Psychol Addict Behav 32(6):605–616. 10.1037/adb0000394 30211583 10.1037/adb0000394

[CR17] DiLalla LF, John SG (2014) Genetic and behavioral influences on received aggression during observed play among unfamiliar preschool-aged peers. Merrill-Palmer Quart 60(2):168–192. 10.13110/merrpalmquar1982.60.2.0168

[CR18] Doumas DM, Midgett A, Johnston AD (2017) Substance use and bullying victimization among middle and high school students: is positive school climate a protective factor? J Addict Offender Couns 38(1):2–15. 10.1002/jaoc.12025

[CR19] Earnshaw VA, Elliott MN, Reisner SL, Mrug S, Windle M, Emery ST, Peskin MF, Schuster MA (2017) Peer victimization, depressive symptoms, and substance use: a longitudinal analysis. Pediatrics 139(6):e20163426. 10.1542/peds.2016-3426 28562268 10.1542/peds.2016-3426 PMC8918284

[CR20] Fergusson DM, Swain-Campbell NR, Horwood LJ (2002) Deviant peer affiliations, crime and substance use: a fixed effects regression analysis. J Abnorm Child Psychol 30(4):419–430. 10.1023/A:1015774125952 12108769 10.1023/a:1015774125952

[CR21] Finkelhor D, Turner HA, Shattuck A, Hamby SL (2015) Prevalence of childhood exposure to violence, crime, and abuse: results from the national survey of children’s exposure to violence. JAMA Pediatr 169(8):746–754. 10.1001/jamapediatrics.2015.0676 26121291 10.1001/jamapediatrics.2015.0676

[CR22] Fowler T, Lifford K, Shelton K, Rice F, Thapar A, Neale MC, McBride A, Van Den Bree MBM (2007) Exploring the relationship between genetic and environmental influences on initiation and progression of substance use. Addiction 102(3):413–422. 10.1111/j.1360-0443.2006.01694.x 17298649 10.1111/j.1360-0443.2006.01694.x PMC1974769

[CR23] Gajos JM, Leban L, Weymouth BB, Cropsey KL (2023) Sex differences in the relationship between early adverse childhood experiences, delinquency, and substance use initiation in high-risk adolescents. J Interpers Violence 38(1–2):311–335. 10.1177/08862605221081927 10.1177/08862605221081927 35466765

[CR24] Gladden, R. M., Vivolo-Kantor, A. M., Hamburger, M. E., & Lumpkin, C. D. (2014) *Bullying surveillance among youths: Uniform definitions for public health and recommended data elements, Version 1.0*. https://stacks.cdc.gov/view/cdc/21596

[CR25] Guerra LM, Romano PS, Samuels SJ, Kass PH (2000) Ethnic differences in adolescent substance initiation sequences. Arch Pediatr Adolesc Med 154(11):1089–1095. 10.1001/archpedi.154.11.1089 11074848 10.1001/archpedi.154.11.1089

[CR26] Guimond F-A, Brendgen M, Correia S, Turgeon L, Vitaro F (2018) The moderating role of peer norms in the associations of social withdrawal and aggression with peer victimization. Dev Psychol 54(8):1519–1527. 10.1037/dev0000539 29927263 10.1037/dev0000539

[CR27] Guy A, Lee K, Wolke D (2019) Comparisons between adolescent bullies, victims, and bully-victims on perceived popularity, social impact, and social preference. Front Psychiat. 10.3389/fpsyt.2019.00868 10.3389/fpsyt.2019.00868 PMC6883422 31824358

[CR28] Hamilton, L., & Seyfrit, C. (1993) Interpreting multinomial logistic regression. *Stata Technical Bulletin*. https://scholars.unh.edu/soc_facpub/457

[CR29] Hanish LD, Guerra NG (2000) The roles of ethnicity and school context in predicting children’s victimization by peers. Am J Community Psychol 28(2):201–223. 10.1023/A:1005187201519 10836091 10.1023/A:1005187201519

[CR31] Hicks BM, Iacono WG, McGue M (2014) Identifying childhood characteristics that underlie premorbid risk for substance use disorders: socialization and boldness. Dev Psychopathol 26(1):141–157. 10.1017/S0954579413000862 24280373 10.1017/S0954579413000862 PMC3946375

[CR32] Huber, P. J. (1967) The behavior of maximum likelihood estimates under nonstandard conditions. In *Proceedings of the Fifth Berkeley Symposium on Mathematical Statistics and Probability*. University of California Press.

[CR33] Hunter MD (2018) State space modeling in an open source, modular, structural equation modeling environment. Struct Equ Modeling 25(2):307–324. 10.1080/10705511.2017.1369354

[CR34] Jackson, K. M., Marceau, K., Colby, S. M., Barnett, N. P., Rogers, M. L., & Hayes, K. L. (2021) Trajectories of early alcohol use milestones: Interrelations among initiation and progression. *Alcoholism: Clinical and Experimental Research.**45*(11): 2294–2308. 10.1111/acer.14723 10.1111/acer.14723 PMC8642286 34585748

[CR35] Jiang Y, Yu C, Zhang W, Bao Z, Zhu J (2016) Peer victimization and substance use in early adolescence: Influences of deviant peer affiliation and parental knowledge. J Child Fam Stud 25(7):2130–2140. 10.1007/s10826-016-0403-z

[CR36] Jimerson, S., Nickerson, A., Mayer, M. J., & Furlong, M. J. (Eds.). (2012) *Handbook of School Violence and School Safety: International Research and Practice* (2nd ed.). Routledge. 10.4324/9780203841372

[CR37] Johansson A, Huhtamäki A, Sainio M, Kaljonen A, Boivin M, Salmivalli C (2022) Heritability of bullying and victimization in children and adolescents: moderation by the KiVa antibullying program. J Clin Child Adolesc Psychol 51(4):505–514. 10.1080/15374416.2020.1731820 32175773 10.1080/15374416.2020.1731820

[CR38] Johnson AL, Villanti AC, Williams V, Rath JM, Vallone DM, Abrams DB, Hedeker D, Mermelstein RJ (2019) Smoking trajectory classes and impact of social smoking identity in two cohorts of US young adults. Emerg Adult 7(4):258–269. 10.1177/2167696818763949 10.1177/2167696818763949 PMC10798807 38250305

[CR39] Keskitalo K, Tuorila H, Spector TD, Cherkas LF, Knaapila A, Silventoinen K, Perola M (2007) Same genetic components underlie different measures of sweet taste preference. Am J Clin Nutr 86(6):1663–1669. 10.1093/ajcn/86.5.1663 18065584 10.1093/ajcn/86.5.1663

[CR40] Keyes KM, Martins SS, Blanco C, Hasin DS (2010) Telescoping and gender differences in alcohol dependence: new evidence from two national surveys. Am J Psychiatry 167(8):969–976. 10.1176/appi.ajp.2009.09081161 20439391 10.1176/appi.ajp.2009.09081161 PMC3767417

[CR41] Khantzian EJ (1985) The self-medication hypothesis of addictive disorders: focus on heroin and cocaine dependence. Am J Psychiatry 142(11):1259–1264. 10.1176/ajp.142.11.1259 3904487 10.1176/ajp.142.11.1259

[CR42] Khantzian EJ (1997) The self-medication hypothesis of substance use disorders: a reconsideration and recent applications. Harv Rev Psychiatry 4(5):231–244. 10.3109/10673229709030550 9385000 10.3109/10673229709030550

[CR43] Klomek AB, Sourander A, Elonheimo H (2015) Bullying by peers in childhood and effects on psychopathology, suicidality, and criminality in adulthood. Lancet Psychiatr 2(10):930–941. 10.1016/S2215-0366(15)00223-0 10.1016/S2215-0366(15)00223-0 26462227

[CR44] Kowalski RM, Limber SP, McCord A (2019) A developmental approach to cyberbullying: prevalence and protective factors. Aggress Violent Beh 45:20–32. 10.1016/j.avb.2018.02.009

[CR45] Kranzler HR, Feinn R, Xu H, Ho BL, Saini D, Nicastro OR, Jacoby A, Toikumo S, Gelernter J, Hartwell EE, Kember RL (2023) Does polygenic risk for substance-related traits predict ages of onset and progression of symptoms? Addiction 118(9):1675–1686. 10.1111/add.16210 37069489 10.1111/add.16210 PMC10525011

[CR46] Kshirsagar VY, Agarwal R, Bavdekar SB (2007) Bullying in schools: prevalence and short-term impact. Indian Pediatr 44(1):25–28 17277427

[CR47] Laroque FM, Boers E, Afzali MH, Conrod PJ (2023) Personality-specific pathways from bullying victimization to adolescent alcohol use: a multilevel longitudinal moderated mediation analysis. Dev Psychopathol 35(3):1454–1467. 10.1017/S0954579421001358 35129105 10.1017/S0954579421001358

[CR48] Lees B, Meredith LR, Kirkland AE, Bryant BE, Squeglia LM (2020) Effect of alcohol use on the adolescent brain and behavior. Pharmacol Biochem Behav 192:172906. 10.1016/j.pbb.2020.172906 32179028 10.1016/j.pbb.2020.172906 PMC7183385

[CR50] Little RJA (1988) A test of missing completely at random for multivariate data with missing values. J Am Stat Assoc 83(404):1198–1202. 10.1080/01621459.1988.10478722

[CR51] Matthews T, Caspi A, Danese A, Fisher HL, Moffitt TE, Arseneault L (2022) A longitudinal twin study of victimization and loneliness from childhood to young adulthood. Dev Psychopathol 34(1):367–377. 10.1017/S0954579420001005 33046153 10.1017/S0954579420001005 PMC8041922

[CR52] McDougall P, Vaillancourt T (2015) Long-term adult outcomes of peer victimization in childhood and adolescence: pathways to adjustment and maladjustment. Am Psychol 70(4):300–310. 10.1037/a0039174 25961311 10.1037/a0039174

[CR53] Miller RN, Fagan AA, Wright EM (2014) The moderating effects of peer and parental support on the relationship between vicarious victimization and substance use. J Drug Issues 44(4):362–380. 10.1177/0022042614526995 25530628 10.1177/0022042614526995 PMC4269271

[CR54] Mlawer F, Hubbard JA, Bookhout MK, Moore CC, Docimo MA, Swift LE, Grassetti SN (2019) Bidirectional relations between internalizing symptoms and peer victimization in late childhood. Soc Dev 28(4):942–959. 10.1111/sode.12371

[CR55] Monks CP, Smith PK, Naylor P, Barter C, Ireland JL, Coyne I (2009) Bullying in different contexts: commonalities, differences and the role of theory. Aggress Violent Beh 14(2):146–156. 10.1016/j.avb.2009.01.004

[CR56] Morales DX, Grineski SE, Collins TW (2019) School bullying, body size, and gender: an intersectionality approach to understanding US children’s bullying victimization. Br J Sociol Educ 40(8):1121–1137. 10.1080/01425692.2019.1646115 33041392 10.1080/01425692.2019.1646115 PMC7542988

[CR57] Mynard H, Joseph S (1997) Bully/victim problems and their association with Eysenck’s personality dimensions in 8 to 13 year-olds. Br J Educ Psychol 67(1):51–54. 10.1111/j.2044-8279.1997.tb01226.x 9114731 10.1111/j.2044-8279.1997.tb01226.x

[CR58] Neale MC, Hunter MD, Pritikin JN, Zahery M, Brick TR, Kirkpatrick RM, Estabrook R, Bates TC, Maes HH, Boker SM (2016) OpenMx 2.0: Extended structural equation and statistical modeling. Psychometrika 81(2):535–549. 10.1007/s11336-014-9435-8 25622929 10.1007/s11336-014-9435-8 PMC4516707

[CR60] Poelen, E. A. P., Derks, E. M., Engels, R. C. M. E., Van Leeuwe, J. F. J., Scholte, R. H. J., Willemsen, G., & Boomsma, D. I. (2008) The relative contribution of genes and environment to alcohol use in early adolescents: Are similar factors related to initiation of alcohol use and frequency of drinking? *Alcoholism: Clinical and Experimental Research.**32*(6): 975–982. 10.1111/j.1530-0277.2008.00657.x 10.1111/j.1530-0277.2008.00657.x 18445102

[CR61] Pulkkinen L, Vaalamo I, Hietala R, Kaprio J, Rose RJ (2003) Peer reports of adaptive behavior in twins and singletons: is twinship a risk or an advantage? Twin Res Hum Genet 6(2):106–118. 10.1375/twin.6.2.106 10.1375/136905203321536236 12723997

[CR62] Rhee SH, Hewitt JK, Young SE, Corley RP, Crowley TJ, Stallings MC (2003) Genetic and environmental influences on substance initiation, use, and problem use in adolescents. Arch Gen Psychiatry 60(12):1256–1264. 10.1001/archpsyc.60.12.1256 14662558 10.1001/archpsyc.60.12.1256

[CR63] Rice KG, FitzGerald DP, Whaley TJ, Gibbs CL (1995) Cross-sectional and longitudinal examination of attachment, separation-individuation, and college student adjustment. J Couns Dev 73(4):463–474. 10.1002/j.1556-6676.1995.tb01781.x

[CR64] Richmond-Rakerd LS, Slutske WS, Lynskey MT, Agrawal A, Madden PAF, Bucholz KK, Heath AC, Statham DJ, Martin NG (2016) Age at first use and later substance use disorder: shared genetic and environmental pathways for nicotine, alcohol, and cannabis. J Abnorm Psychol 125(7):946–959. 10.1037/abn0000191 27537477 10.1037/abn0000191 PMC5061603

[CR96] Roche DJO, Bujarski S, Green R, Hartwell EE, Leventhal AM, Ray LA (2019) Alcohol, tobacco, and marijuana consumption is associated with increased odds of same-day substance co- and tri-use. Drug Alcohol Depend 200:40–49. 10.1016/j.drugalcdep.2019.02.035 31085377 10.1016/j.drugalcdep.2019.02.035 PMC6675401

[CR65] Rodkin PC, Berger C (2008) Who bullies whom? Social status asymmetries by victim gender. Int J Behav Dev 32(6):473–485. 10.1177/0165025408093667

[CR66] Rospenda KM, Richman JA, Wolff JM, Burke LA (2013) Bullying victimization among college students: negative consequences for alcohol use. J Addict Dis 32(4):325–342. 10.1080/10550887.2013.849971 24325767 10.1080/10550887.2013.849971 PMC3861792

[CR67] Rudolph KD, Lansford JE, Agoston AM, Sugimura N, Schwartz D, Dodge KA, Pettit GS, Bates JE (2014) Peer victimization and social alienation: predicting deviant peer affiliation in middle school. Child Dev 85(1):124–139. 10.1111/cdev.12112 23621796 10.1111/cdev.12112 PMC3732545

[CR68] Sapouna M, Wolke D (2013) Resilience to bullying victimization: the role of individual, family and peer characteristics. Child Abuse Negl 37(11):997–1006. 10.1016/j.chiabu.2013.05.009 23809169 10.1016/j.chiabu.2013.05.009

[CR69] Scarr S, McCartney K (1983) How people make their own environments: a theory of genotype → environment effects. Child Dev 54(2):424–435. 10.2307/1129703 6683622 10.1111/j.1467-8624.1983.tb03884.x

[CR70] Scheithauer H, Hayer T, Petermann F, Jugert G (2006) Physical, verbal, and relational forms of bullying among German students: age trends, gender differences, and correlates. Aggressive Behav 32(3):261–275. 10.1002/ab.20128

[CR71] Schumann L, Craig W, Rosu A (2014) Power differentials in bullying: individuals in a community context. J Interpers Viol 29(5):846–865. 10.1177/0886260513505708 10.1177/0886260513505708 24203985

[CR72] Shelton K, Lifford K, Fowler T, Rice F, Neale M, Harold G, Thapar A, van den Bree M (2007) The association between conduct problems and the initiation and progression of marijuana use during adolescence: a genetic analysis across time. Behav Genet 37(2):314–325. 10.1007/s10519-006-9124-1 17131199 10.1007/s10519-006-9124-1 PMC1824713

[CR73] Smith RG, Gross AM (2006) Bullying: prevalence and the effect of age and gender. Child Fam Behav Ther 28(4):13–37. 10.1300/J019v28n04_02

[CR74] Smithyman TF, Fireman GD, Asher Y (2014) Long-term psychosocial consequences of peer victimization: from elementary to high school. Sch Psychol Q 29(1):64–76. 10.1037/spq0000053 24708289 10.1037/spq0000053

[CR75] Stocker C, Dunn J (1990) Sibling relationships in childhood: links with friendships and peer relationships. Br J Dev Psychol 8(3):227–244. 10.1111/j.2044-835X.1990.tb00838.x

[CR76] Storch EA, Ledley DR (2005) Peer victimization and psychosocial adjustment in children: current knowledge and future directions. Clin Pediatr 44(1):29–38. 10.1177/000992280504400103 10.1177/000992280504400103 15678228

[CR77] Sullivan TN, Farrell AD, Kliewer W (2006) Peer victimization in early adolescence: association between physical and relational victimization and drug use, aggression, and delinquent behaviors among urban middle school students. Dev Psychopathol 18(1):119–137. 10.1017/S095457940606007X 16478555 10.1017/S095457940606007X

[CR78] Tabachnick, B. G., & Fidell, L. S. (2018). *Using Multivariate Statistics*. Pearson.

[CR79] TenEyck M, Barnes JC (2015) Examining the impact of peer group selection on self-reported delinquency: a consideration of active gene–environment correlation. Crim Justice Behav 42(7):741–762. 10.1177/0093854814563068

[CR80] Tharp-Taylor S, Haviland A, D’Amico EJ (2009) Victimization from mental and physical bullying and substance use in early adolescence. Addict Behav 34(6):561–567. 10.1016/j.addbeh.2009.03.012 19398162 10.1016/j.addbeh.2009.03.012 PMC2707251

[CR81] Thorpe HHA, Hamidullah S, Jenkins BW, Khokhar JY (2020) Adolescent neurodevelopment and substance use: receptor expression and behavioral consequences. Pharmacol Ther 206:107431. 10.1016/j.pharmthera.2019.107431 31706976 10.1016/j.pharmthera.2019.107431

[CR82] Van Ryzin MJ, Dishion TJ (2014) Adolescent deviant peer clustering as an amplifying mechanism underlying the progression from early substance use to late adolescent dependence. J Child Psychol Psychiatry 55(10):1153–1161. 10.1111/jcpp.12211 24673521 10.1111/jcpp.12211 PMC4133330

[CR83] Vannucci A, Fagle TR, Simpson EG, Ohannessian CM (2021) Perceived family and friend support moderate pathways from peer victimization to substance use in early-adolescent girls and boys: a moderated-mediation analysis. J Early Adolesc 41(1):128–166. 10.1177/0272431620931187

[CR84] Veenstra R, Lindenberg S, Oldehinkel AJ, De Winter AF, Verhulst FC, Ormel J (2005) Bullying and victimization in elementary schools: a comparison of bullies, victims, bully/victims, and uninvolved preadolescents. Dev Psychol 41(4):672–682. 10.1037/0012-1649.41.4.672 16060813 10.1037/0012-1649.41.4.672

[CR85] Veldkamp SAM, Boomsma DI, de Zeeuw EL, van Beijsterveldt CEM, Bartels M, Dolan CV, van Bergen E (2019) Genetic and environmental influences on different forms of bullying perpetration, bullying victimization, and their co-occurrence. Behav Genet 49(5):432–443. 10.1007/s10519-019-09968-5 31502010 10.1007/s10519-019-09968-5 PMC6768918

[CR86] Verweij KJH, Zietsch BP, Lynskey MT, Medland SE, Neale MC, Martin NG, Boomsma DI, Vink JM (2010) Genetic and environmental influences on cannabis use initiation and problematic use: a meta-analysis of twin studies. Addiction 105(3):417–430. 10.1111/j.1360-0443.2009.02831.x 20402985 10.1111/j.1360-0443.2009.02831.x PMC2858354

[CR87] Vieno A, Gini G, Santinello M (2011) Different forms of bullying and their association to smoking and drinking behavior in italian adolescents. J Sch Health 81(7):393–399. 10.1111/j.1746-1561.2011.00607.x 21668879 10.1111/j.1746-1561.2011.00607.x

[CR88] Walters GD, Espelage DL (2018) Exploring the victimization-early substance misuse relationship: in search of moderating and mediating effects. Child Abuse Negl 81:354–365. 10.1016/j.chiabu.2018.05.006 29793150 10.1016/j.chiabu.2018.05.006

[CR89] Wang J, Iannotti RJ, Nansel TR (2009) School bullying among adolescents in the United States: physical, verbal, relational, and cyber. J Adolesc Health 45(4):368–375. 10.1016/j.jadohealth.2009.03.021 19766941 10.1016/j.jadohealth.2009.03.021 PMC2751860

[CR90] Wei H-S, Chen J-K (2008) Social withdrawal, peer rejection, and peer victimization in taiwanese middle school students. J Sch Violence 8(1):18–28. 10.1080/15388220802067755

[CR91] Weiss JW, Mouttapa M, Cen S, Johnson CA, Unger J (2011) Longitudinal effects of hostility, depression, and bullying on adolescent smoking initiation. J Adolesc Health 48(6):591–596. 10.1016/j.jadohealth.2010.09.012 21575819 10.1016/j.jadohealth.2010.09.012 PMC3096829

[CR92] White H (1980) A heteroskedasticity-consistent covariance matrix estimator and a direct test for heteroskedasticity. Econometrica 48(4):817–838. 10.2307/1912934

[CR93] Wu HZ, Temple JR, Shokar NK, Nguyen-Oghalai TU, Grady JJ (2010) Differential racial/ethnic patterns in substance use initiation among young, low-income women. Am J Drug Alcohol Abuse 36(2):123–129. 10.3109/00952991003718072 20337510 10.3109/00952991003718072

[CR94] Zellers SM, Iacono WG, McGue M, Vrieze S (2022) Developmental and etiological patterns of substance use from adolescence to middle age: a longitudinal twin study. Drug Alcohol Depend 233:109378. 10.1016/j.drugalcdep.2022.109378 35248999 10.1016/j.drugalcdep.2022.109378 PMC8957537

[CR95] Zilberman M, Tavares H, El-Guebaly N (2004) Gender similarities and differences. J Addict Dis 22(4):61–74. 10.1300/J069v22n04_06 10.1300/j069v22n04_06 14723478

