# Supplementary material for: Peer Victimization in Childhood and Timing of Substance Use Initiation: Evidence from a Twin Study
Source: Behav Genet. 2025 Jul 12;55(4):270–88. doi: 10.1007/s10519-025-10222-4 (PMC12325545; doi:10.1007/s10519-025-10222-4)
Supplement: Supplementary file 1 — Supplementary file1 (DOCX 709 kb) [file 10519_2025_10222_MOESM1_ESM.docx]

**Supplementary Materials for Peer victimization in childhood and timing of substance use initiation:**

**Evidence from a twin study**

***Supplementary Material S1 – Substance Use Initiation Data from RFAB Team***

| *Table S1:* Proportion of Early Initiators (by age 14-16 years at Wave 3) | | | |
| --- | --- | --- | --- |
|  | **Cigarette** | **Alcohol** | **Marijuana** |
| **%** | 0.150 | 0.181 | 0.105 |
| **N** | 173 | 155 | 119 |

| *Table S2:* Substance Use Initiation Categories | | | |
| --- | --- | --- | --- |
|  | **Cigarette**  **(N) %** | **Alcohol**  **(N) %** | **Marijuana**  **(N) %** |
| ***Never*** | (410) 54.59% | (209) 40.12% | (369) 53.32% |
| ***Early (W3)*** | (173) 23.04% | (155) 29.75% | (119) 17.20% |
| ***Mid (W4)*** | (120) 15.98% | (80) 15.36% | (145) 20.95% |
| ***Late (W5)*** | (48) 6.39% | (77) 14.78% | (59) 8.53% |

| *Table S3:* Age at Censoring and Initiation Age | | |
| --- | --- | --- |
| Ages in Years | **Age at Censoring**  **(N) Mean ± SD** | **Age at Substance Use**  **(N) Mean ± SD** |
| ***Cigarette*** | (1104) 15.4 ± 3.8 | (458) 14.7 ± 3.1 |
| ***Alcohol*** | (808) 14.2 ± 3.8 | (662) 15.6 ± 2.4 |
| ***Marijuana*** | (1082) 15.2 ± 3.8 | (480) 15.1 ± 2.0 |

***Supplementary Material S2 – Bar Chart of Timing of Substance Initiation of Children Who Experienced Victimization or Not***

*Figure S2.1.* Percentage of adolescents in categories of timing of alcohol initiation


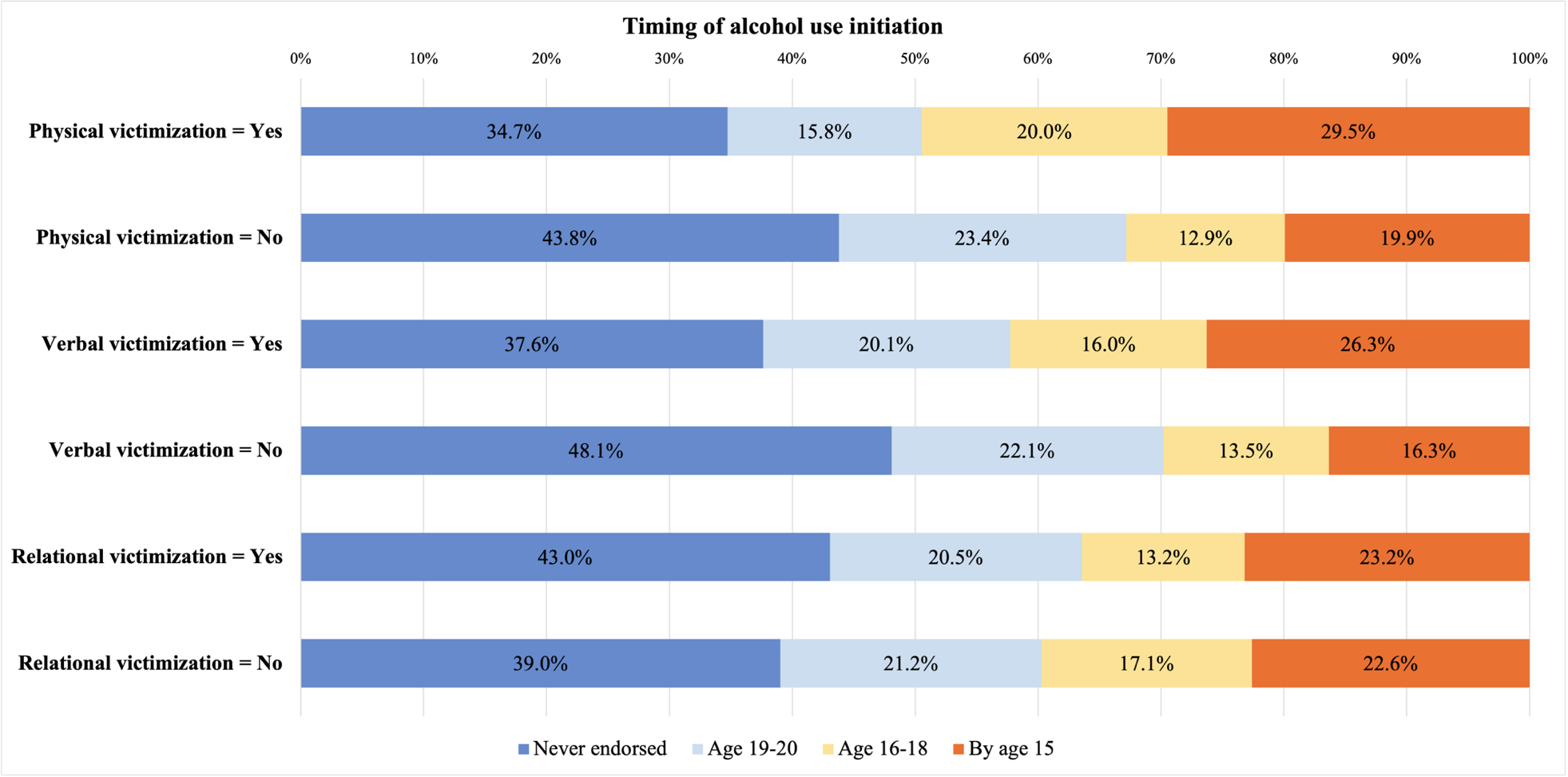


*Note:* This bar chart is only for displaying the tendency of timing of substance use initiation in participants experienced/not experienced victimization to readers, and not for any steps in analytic plans.

*Figure S2.2.* Percentage of adolescents in categories of timing of cigarette initiation


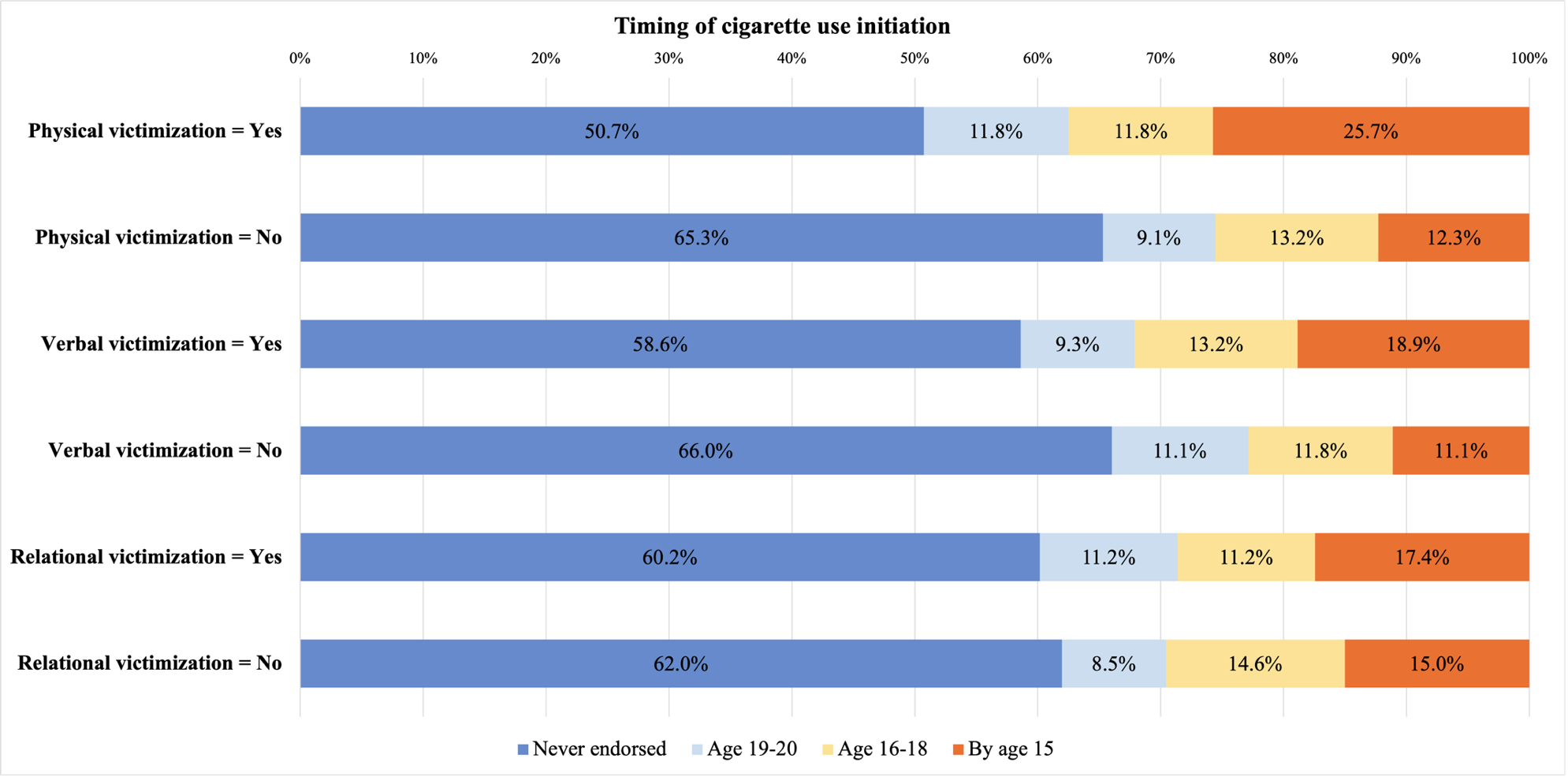


*Figure S2.3.* Percentage of adolescents in categories of timing of marijuana initiation


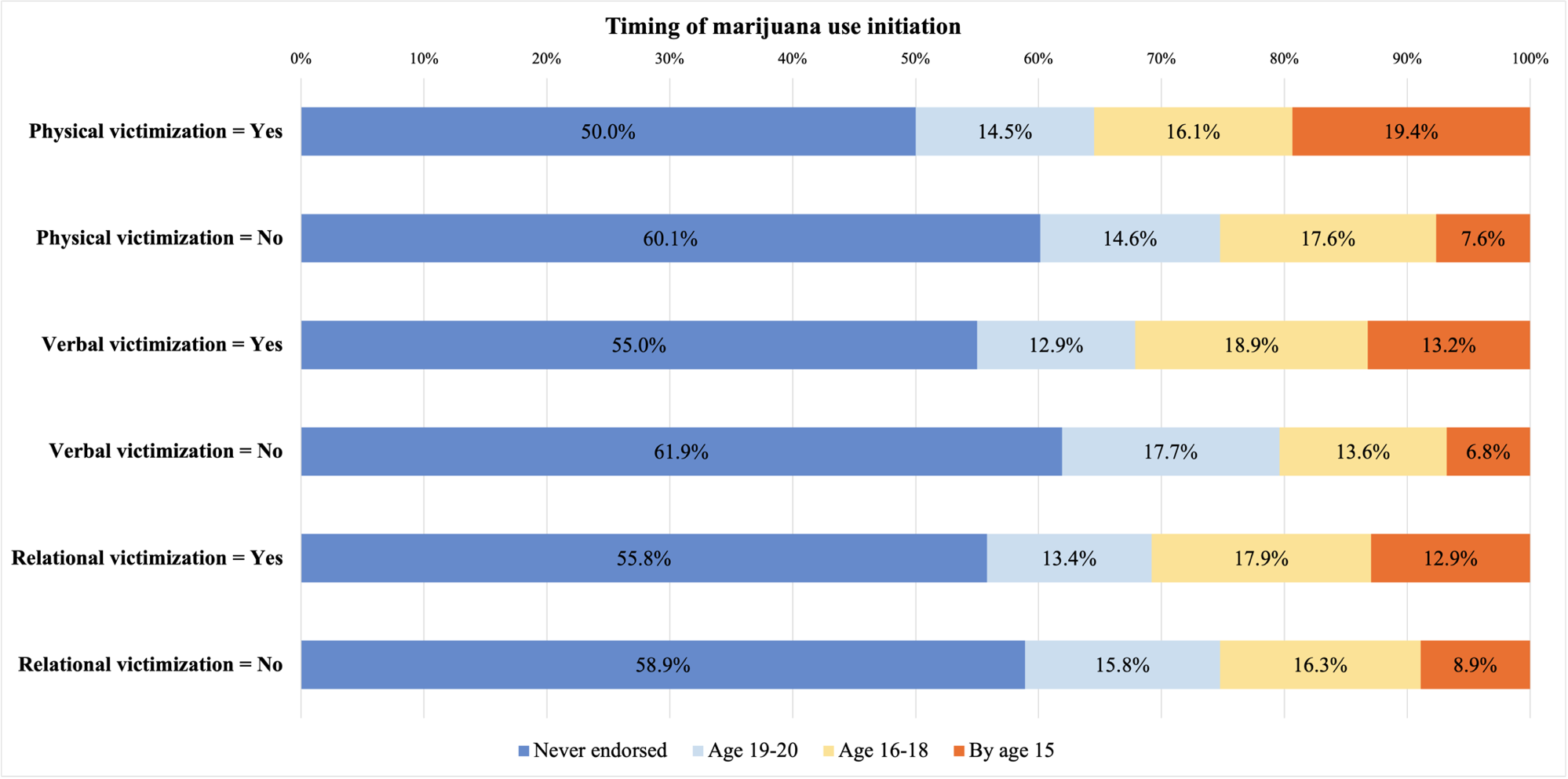


***Supplementary Material S3 – Results of Sensitivity Analysis Using the Sample of 237 Twin Pairs***

*Table S3.1.* Sociodemographic Characteristics of Twins in Sensitivity Analysis

| **Characteristic** | ***N*** | ***%*** |
| --- | --- | --- |
| *Sex* |  | |
| Female | 271 | 57.2 |
| Male | 203 | 42.8 |
| *Race/Ethnicity* |  | |
| Caucasian | 148 | 31.2 |
| Hispanic | 158 | 33.3 |
| Black | 60 | 12.7 |
| Asian | 26 | 5.5 |
| Multiracial | 82 | 17.3 |
| *Zygosity* |  | |
| MZ males | 47 (pairs) | 19.8 |
| MZ females | 66 (pairs) | 27.8 |
| DZ males | 29 (pairs) | 12.2 |
| DZ females | 44 (pairs) | 18.6 |
| DZ opposite sex | 51 (pairs) | 21.5 |

*Table S3.2.* Descriptive Results for Peer Victimization and Timing of Substance Initiation in Sensitivity Analysis

| **Variable** | ***N (Individuals)*** | ***M*** | ***SD*** |  | **Level** | | | | | | | | | | |
| --- | --- | --- | --- | --- | --- | --- | --- | --- | --- | --- | --- | --- | --- | --- | --- |
|  |  |  |  | **Skewness** | **1** | | **2** | | | **3** | | **4** | | |  |
|  |  |  |  |  | ***N*** | ***%*** | | ***N*** | ***%*** | ***N*** | ***%*** | | ***N*** | ***%*** | |
| Physical victimization | 472 | 1.63 | 1.15 | 1.87 | / | / | | / | / | / | / | | / | / | |
| Verbal victimization | 474 | 2.10 | 1.18 | .98 | / | / | | / | / | / | / | | / | / | |
| Relational victimization | 473 | 2.11 | 1.31 | .94 | / | / | | / | / | / | / | | / | / | |
| Timing of cigarette use initiation | 455 | / | / | .93 | 278 | 61.1 | | 45 | 9.9 | 58 | 12.7 | | 74 | 16.3 | |
| Timing of alcohol use initiation | 298 | / | / | .42 | 123 | 41.3 | | 62 | 20.8 | 45 | 15.1 | | 68 | 22.8 | |
| Timing of marijuana use initiation | 427 | / | / | .91 | 245 | 57.4 | | 62 | 14.5 | 73 | 17.1 | | 47 | 11.0 | |

*Note:* Due to data skewness, physical victimization was Box-Cox transformed, with the skewness after transformation being .68. For timing of substance use initiation variables, Level 1 = Never endorsed by the final assessment: if the child/adolescent never reported ever use in any wave; Level 2 = Late initiation: if the child/adolescent reported the first ever use at age 19-20 (wave 5); Level 3 = Mid initiation: if the child/adolescent reported the first ever use at age 16-18 (wave 4); Level 4 = Early initiation: if the child/adolescent reported the first ever use at or before age 15 (wave 1-3).

*Table S3.3.* Mixed-Effect Model Results for Sex Differences in Peer Victimization and Timing of Substance Use Initiation

|  |  | ***N*** | ***Mean*** | ***SD*** | ***F*** | ***p*** |
| --- | --- | --- | --- | --- | --- | --- |
| Physical Victimization | Male | 203 | 1.77 | 1.25 | 1.51 | .22 |
|  | Female | 269 | 1.62 | 1.11 |  |  |
| Verbal victimization | Male | 203 | 2.14 | 1.18 | .00 | .98 |
|  | Female | 271 | 2.12 | 1.16 |  |  |
| Relational victimization | Male | 203 | 2.27 | 1.38 | .75 | .39 |
|  | Female | 270 | 2.10 | 1.32 |  |  |
| Timing of cigarette use initiation | Male | 193 | 2.02 | 1.24 | 1.42 | .24 |
|  | Female | 262 | 1.97 | 1.25 |  |  |
| Timing of alcohol use initiation | Male | 134 | 2.36 | 1.23 | .82 | .37 |
|  | Female | 164 | 2.39 | 1.25 |  |  |
| Timing of marijuana use initiation | Male | 186 | 2.01 | 1.15 | 1.17 | .28 |
|  | Female | 241 | 1.97 | 1.16 |  |  |

*Table S3.4.* Mixed-Effect Model Results for Racial/Ethnic Differences in Peer Victimization and Timing of Substance Use Initiation

| **Variable** | ***F* (*df1*, *df2*)** | ***p*-value** | **Post Hoc Comparisons** |
| --- | --- | --- | --- |
| Physical victimization | .89 (4, 237) | .47 | / |
| Verbal Victimization | 1.18 (4, 237) | .32 | / |
| Relational Victimization | 1.74 (4, 237) | .14 | / |
| Timing of cigarette use initiation | .31 (4, 237) | .87 | / |
| Timing of alcohol use initiation | 1.38 (4, 237) | .24 | / |
| Timing of marijuana use initiation | 1.55 (4, 237) | .19 | / |

*Note:* M diff = difference between means. Higher score on victimization represents more victimization experiences at age 9-10. Higher score on timing of substance use initiation represents earlier initiation.

*Table S3.5.* Multinomial Logistic Regressions Between Childhood Victimization and Timing of Substance Use Initiation

(Reference Group = Never Endorsed by the Final Assessment)

|  | | Physical Victimization | Verbal Victimization | Relational Victimization |
| --- | --- | --- | --- | --- |
| *RRR (95% CI)* | | | | |
| Timing of cigarette use initiation | 19-20yo | 4.13 (.53, 32.03) | .74 (.48, 1.14) | **1.37 (1.03, 1.82)** |
|  | 16-18yo | .85 (.13, 5.59) | 1.14 (.85, 1.53) | 1.08 (.82, 1.43) |
|  | <15yo | **6.18 (1.16, 32.96)** | 1.28 (.93, 1.75) | .93 (.70, 1.22) |
| Timing of alcohol use initiation | 19-20yo | .77 (.10, 5.79) | 1.07 (.73, 1.57) | .89 (.68, 1.17) |
|  | 16-18yo | 6.62 (.79, 55.38) | 1.24 (.81, 1.88) | **.60 (.40, .89)** |
|  | <15yo | 1.64 (.23, 11.52) | 1.32 (.90, 1.93) | .89 (.64, 1.23) |
| Timing of marijuana use initiation | 19-20yo | 2.10 (.37, 11.95) | 1.02 (.72, 1.45) | .93 (.70, 1.23) |
|  | 16-18yo | .56 (.09, 3.56) | 1.24 (.91, 1.67) | 1.08 (.84, 1.40) |
|  | <15yo | 5.24 (.66, 41.39) | 1.33 (.92, 1.92) | 1.01 (.76, 1.35) |

*Note:* For timing of substance use initiation variables, Level 1 = Never endorsed by the final assessment: if the child/adolescent never reported ever use in any wave; Level 2 = Late initiation: if the child/adolescent reported the first ever use at age 19-20 (wave 5); Level 3 = Mid initiation: if the child/adolescent reported the first ever use at age 16-18 (wave 4); Level 4 = Early initiation: if the child/adolescent reported the first ever use at or before age 15 (wave 1-3). Bold values represent significant results (i.e., 95%CI does not include 1.0). The number of observations is 453 for cigarette initiation models, 296 for alcohol initiation models, and 425 for marijuana initiation models.

(Reference Group = Initiated at age 19-20)

|  |  | Physical Victimization | Verbal Victimization | Relational Victimization |
| --- | --- | --- | --- | --- |
| *RRR (95% CI)* | | | | |
| Timing of cigarette use initiation | Never endorsed | .24 (.03, 1.88) | 1.35 (.88, 2.08) | **.73 (.55, .97)** |
|  | 16-18yo | .21 (.02, 2.53) | 1.54 (.96, 2.48) | .79 (.56, 1.11) |
|  | <15yo | 1.50 (.14, 15.68) | **1.73 (1.06, 2.80)** | **.68 (.47, .98)** |
| Timing of alcohol use initiation | No use by 21yo | 1.30 (.17, 9.74) | .93 (.64, 1.36) | 1.12 (.85, 1.48) |
|  | 16-18yo | 8.59 (.78, 95.16) | 1.15 (.74, 1.78) | .67 (.44, 1.01) |
|  | <15yo | 2.13 (.24, 19.20) | 1.23 (.83, 1.82) | 1.00 (.72, 1.39) |
| Timing of marijuana use initiation | No use by 21yo | .48 (.08, 2.70) | .98 (.69, 1.40) | 1.08 (.82, 1.42) |
|  | 16-18yo | .27 (.03, 2.22) | 1.21 (.81, 1.81) | 1.17 (.84, 1.62) |
|  | <15yo | 2.49 (.26, 24.14) | 1.30 (.83, 2.04) | 1.09 (.76, 1.56) |

(Reference Group = Initiated at age 16-18)

|  |  | Physical Victimization | Verbal Victimization | Relational Victimization |
| --- | --- | --- | --- | --- |
| *RRR (95% CI)* | | | | |
| Timing of cigarette use initiation | No use by 21yo | 1.17 (.18, 7.66) | .88 (.65, 1.18) | .93 (.70, 1.23) |
|  | 19-20yo | 4.84 (.40, 59.14) | .65 (.40, 1.04) | 1.27 (.90, 1.77) |
|  | <15yo | 7.23 (.80, 65.06) | 1.12 (.78, 1.62) | .86 (.60, 1.22) |
| Timing of alcohol use initiation | No use by 21yo | .15 (.02, 1.26) | .81 (.53, 1.23) | **1.68 (1.13, 2.50)** |
|  | 19-20yo | .12 (.01, 1.29) | .87 (.56, 1.34) | 1.49 (.99, 2.26) |
|  | <15yo | .25 (.03, 2.37) | 1.07 (.70, 1.62) | 1.49 (.99, 2.23) |
| Timing of marijuana use initiation | No use by 21yo | 1.78 (.28, 11.24) | .81 (.60, 1.09) | .92 (.72, 1.19) |
|  | 19-20yo | 3.74 (.45, 31.06) | .83 (.55, 1.23) | .86 (.62, 1.19) |
|  | <15yo | 9.31 (.92, 94.61) | 1.08 (.72, 1.60) | .93 (.66, 1.31) |

*Table S3.6.* Phenotypic Correlation Matrix for Peer Victimization and Substance Initiation Variables

| Variable | 1 | 2 | 3 | 4 | 5 | 6 |
| --- | --- | --- | --- | --- | --- | --- |
| 1 Physical Victimization | 1 |  |  |  |  |  |
| 2 Verbal Victimization | .55*** | 1 |  |  |  |  |
| 3 Relational Victimization | .38*** | .55*** | 1 |  |  |  |
| 4 Timing of Cigarette Use Initiation | .17*** | .16*** | .06 | 1 |  |  |
| 5 Timing of Alcohol Use Initiation | .13* | .10 | -.01 | .72*** | 1 |  |
| 6 Timing of Marijuana Use Initiation | .14** | .14** | .09 | .77*** | .74*** | 1 |

*Note:* .08< *^+^p* <.05, **p* < .05, ***p* < .01, ****p* < .001

*Table S3.7.* Twin Correlation Matrix for Peer Victimization and Timing of Substance Use Initiation Variables

|  | 1 Physical Victimization | 2 Verbal Victimization | 3 Relational Victimization | 4 Timing of Cigarette Use Initiation | 5 Timing of Alcohol Use Initiation | 6 Timing of Marijuana Use Initiation |
| --- | --- | --- | --- | --- | --- | --- |
| *MZ vs. DZ* | | | | | | |
| MZ | .31 | .47 | .21 | .80 | .88 | .81 |
| DZ | .18 | .08 | .12 | .53 | .62 | .63 |
| *MZF vs. DZF* | | | | | | |
| MZF | .39 | .44 | .26 | .82 | .88 | .82 |
| DZF | .13 | .03 | -.04 | .66 | .84 | .76 |
| *MZM vs. DZM* | | | | | | |
| MZM | .20 | .53 | .15 | .78 | .89 | .80 |
| DZM | .32 | .23 | .33 | .37 | .74 | .56 |
| *DZOS* | | | | | | |
| DZOS | .15 | .09 | .19 | .51 | .45 | .58 |

*Note:* MZF = MZ female twin pairs, DZF = DZ female twin pairs, MZM = MZ male twin pairs, DZM = DZ male twin pairs, DZOS = DZ opposite-sex pairs.

*Table S3.8.* Cross-twin Cross-trait Correlation Matrix of Main Variables for MZ and DZ Twin Pairs (DZ Correlations Below the Diagonal)

|  | T1 VV | T1 PV | T1 RV | T1 ToC | T1 ToA | T1 ToM | T2 VV | T2 PV | T2 RV | T2 ToC | T2 ToA | T2 ToM |
| --- | --- | --- | --- | --- | --- | --- | --- | --- | --- | --- | --- | --- |
| T1 VV | 1.00 | .50 | .49 | .21 | .07 | .22 | .47 | .32 | .36 | .22 | .26 | .25 |
| T1 PV | .48 | 1.00 | .36 | .17 | .15 | .13 | .32 | .31 | .32 | .21 | .44 | .18 |
| T1 RV | .50 | .31 | 1.00 | -.06 | -.27 | -.13 | .15 | .27 | .21 | -.06 | .01 | -.13 |
| T1 ToC | .03 | .12 | -.02 | 1.00 | .68 | .83 | .34 | .23 | .11 | .80 | .67 | .70 |
| T1 ToA | -.08 | -.03 | -.15 | .72 | 1.00 | .77 | .27 | .20 | .09 | .72 | .88 | .71 |
| T1 ToM | .04 | .10 | .00 | .73 | .64 | 1.00 | .33 | .21 | .17 | .78 | .70 | .81 |
| T2 VV | .08 | .57 | .07 | -.01 | -.10 | -.05 | 1.00 | .59 | .60 | .38 | .38 | .40 |
| T2 PV | .10 | .18 | .10 | .11 | .08 | -.04 | .65 | 1.00 | .33 | .23 | .24 | .26 |
| T2 RV | .08 | .14 | .12 | .07 | -.09 | .02 | .60 | .51 | 1.00 | .15 | .21 | .28 |
| T2 ToC | -.01 | .01 | -.03 | .53 | .48 | .38 | .01 | .14 | .12 | 1.00 | .80 | .82 |
| T2 ToA | -.03 | .08 | -.10 | .50 | .62 | .49 | .04 | .15 | .07 | .69 | 1.00 | .78 |
| T2 ToM | .04 | .12 | -.03 | .52 | .40 | .63 | -.02 | .09 | .17 | .74 | .78 | 1.00 |

*Note:* T1 = Twin 1; T2 = Twin 2; VV = Verbal Victimization; PV = Physical Victimization; RV = Relational Victimization; ToC = Timing of cigarette use initiation; ToA = Timing of alcohol use initiation; ToM = Timing of marijuana use initiation

*Table S3.9.* Within-twin Concordance for Timing of Substance Use Initiation Variables

(Timing of cigarette use initiation)

|  |  | MZ Twin 1 | | | |
| --- | --- | --- | --- | --- | --- |
|  |  | Level 1 | Level 2 | Level 3 | Level 4 |
| MZ Twin 2 | Level 1 | 66 (61.11%) | 2 (1.85%) | 3 (2.78%) | 1 (.93%) |
|  | Level 2 | 2 (1.85%) | 3 (2.78%) | 1 (.93%) | 0 (.00%) |
|  | Level 3 | 2 (1.85%) | 0 (.00%) | 9 (8.33%) | 3 (2.78%) |
|  | Level 4 | 0 (.00%) | 4 (3.70%) | 2 (1.85%) | 10 (9.26%) |

*Note:* For timing of substance use initiation variables, Level 1 = Never endorsed by the final assessment: if the child/adolescent never reported ever use in any wave; Level 2 = Late initiation: if the child/adolescent reported the first ever use at age 19-20 (wave 5); Level 3 = Mid initiation: if the child/adolescent reported the first ever use at age 16-18 (wave 4); Level 4 = Early initiation: if the child/adolescent reported the first ever use at or before age 15 (wave 1-3).

|  |  | DZ Twin 1 | | | |
| --- | --- | --- | --- | --- | --- |
|  |  | Level 1 | Level 2 | Level 3 | Level 4 |
| DZ Twin 2 | Level 1 | 49 (43.36%) | 8 (7.08%) | 5 (4.42%) | 4 (3.54%) |
|  | Level 2 | 4 (3.54%) | 5 (4.42%) | 0 (.00%) | 0 (.00%) |
|  | Level 3 | 5 (4.42%) | 6 (5.31%) | 3 (2.65%) | 1 (.88%) |
|  | Level 4 | 5 (4.42%) | 1 (.88%) | 5 (4.42%) | 12 (10.62%) |

(Timing of alcohol use initiation)

|  |  | MZ Twin 1 | | | |
| --- | --- | --- | --- | --- | --- |
|  |  | Level 1 | Level 2 | Level 3 | Level 4 |
| MZ Twin 2 | Level 1 | 24 (42.11%) | 4 (7.02%) | 1 (1.75%) | 0 (.00%) |
|  | Level 2 | 2 (3.51%) | 4 (7.02%) | 3 (5.26%) | 0 (.00%) |
|  | Level 3 | 0 (.00%) | 1 (1.75%) | 4 (7.02%) | 1 (1.75%) |
|  | Level 4 | 0 (.00%) | 1 (1.75%) | 2 (3.51%) | 10 (17.54%) |

|  |  | DZ Twin 1 | | | |
| --- | --- | --- | --- | --- | --- |
|  |  | Level 1 | Level 2 | Level 3 | Level 4 |
| DZ Twin 2 | Level 1 | 10 (20.41%) | 2 (4.08%) | 4 (8.16%) | 1 (2.04%) |
|  | Level 2 | 5 (10.20%) | 4 (8.16%) | 1 (2.04%) | 0 (.00%) |
|  | Level 3 | 0 (.00%) | 0 (.00%) | 6 (12.24%) | 2 (4.08%) |
|  | Level 4 | 1 (2.04%) | 2 (4.08%) | 2 (4.08%) | 9 (18.37%) |

(Timing of marijuana use initiation)

|  |  | MZ Twin 1 | | | |
| --- | --- | --- | --- | --- | --- |
|  |  | Level 1 | Level 2 | Level 3 | Level 4 |
| MZ Twin 2 | Level 1 | 53 (56.38%) | 3 (3.19%) | 0 (.00%) | 0 (.00%) |
|  | Level 2 | 3 (3.19%) | 5 (5.32%) | 1 (1.06%) | 0 (.00%) |
|  | Level 3 | 6 (6.38%) | 3 (3.19%) | 10 (10.64%) | 1 (1.06%) |
|  | Level 4 | 0 (.00%) | 1 (1.06%) | 2 (2.13%) | 6 (6.38%) |

|  |  | DZ Twin 1 | | | |
| --- | --- | --- | --- | --- | --- |
|  |  | Level 1 | Level 2 | Level 3 | Level 4 |
| DZ Twin 2 | Level 1 | 39 (37.50%) | 6 (5.77%) | 6 (5.77%) | 0 (.00%) |
|  | Level 2 | 7 (6.73%) | 9 (8.65%) | 2 (1.92%) | 1 (.96%) |
|  | Level 3 | 5 (4.81%) | 4 (3.85%) | 7 (6.73%) | 2 (1.92%) |
|  | Level 4 | 1 (.96%) | 3 (2.88%) | 3 (2.88%) | 9 (8.65%) |

*Table S3.10.* Univariate Twin Model Parameter Estimates and Fit Statistics

| **Model** | **A** | **C (D)** | **E** | ***AIC*** | ***BIC*** | ***-2LL*** | ***p*** |
| --- | --- | --- | --- | --- | --- | --- | --- |
| *Verbal Victimization* | | | | | | | |
| ADE | .00 [.00, .49] | .43 [.00, .55] | .57 [.45, .72] | 1487.04 | 1489.05 | 1466.06 | - |
| **AE** | **.40 [.25, .53]** | **-** | **.60 [.47, .75]** | **1486.70** | **1488.59** | **1467.91** | **.17** |
| E | - | - | 1.00 [1.00, 1.00] | 1510.47 | 1512.22 | 1493.84 | <.001 |
| *Physical Victimization* | | | | | | | |
| ACE | .28 [.00, .44] | .02 [.00, .32] | .70 [.56, .87] | -197.46 | -195.45 | -218.43 | - |
| **AE** | **.31 [.15, .44]** | **-** | **.69 [.56, .85]** | **-199.63** | **-197.74** | **-218.42** | **.92** |
| **CE** | **-** | **.23 [.10, .35]** | **.77 [.65, .90]** | **-198.27** | **-196.38** | **-217.06** | **.24** |
| E | - | - | 1.00 [1.00, 1.00] | -187.77 | -186.02 | -204.40 | <.001 |
| *Relational Victimization* | | | | | | | |
| ACE | .18 [.00, .34] | .02 [.00, .26] | .81 [.66, .97] | 1607.87 | 1609.88 | 1586.90 | - |
| **AE** | **.20 [.04, .34]** | **-** | **.80 [.66, .96]** | **1605.70** | **1607.59** | **1586.90** | **.93** |
| **CE** | **-** | **.15 [.02, .27]** | **.85 [.73, .98]** | **1606.17** | **1608.06** | **1587.37** | **.49** |
| E | - | - | 1.00 [1.00, 1.00] | 1609.41 | 1611.16 | 1592.77 | .05 |
| *Timing of cigarette use initiation* | | | | | | | |
| ACE | .62 [.37, .84] | .17 [.00, .40] | .21 [.15, .28] | 1274.61 | 1276.62 | 1253.64 | - |
| **AE** | **.80 [.73, .85]** | **-** | **.20 [.15, .27]** | **1273.80** | **1275.69** | **1255.01** | **.24** |
| CE | - | .62 [.54, .70] | .38 [.30, .46] | 1295.40 | 1297.30 | 1276.61 | <.001 |
| E | - | - | 1.00 [1.00, 1.00] | 1399.47 | 1401.22 | 1382.83 | <.001 |
| *Timing of alcohol use initiation* | | | | | | | |
| ACE | .54 [.25, .88] | .31 [.00, .57] | .15 [.10, .23] | 831.11 | 833.12 | 810.14 | - |
| **AE** | **.85 [.78, .90]** | **-** | **.15 [.10, .22]** | **831.61** | **833.50** | **812.82** | **.10** |
| CE | - | .72 [.62, .79] | .28 [.21, .38] | 843.06 | 844.95 | 824.27 | <.001 |
| E | - | - | 1.00 [1.00, 1.00] | 922.21 | 923.97 | 905.58 | <.001 |
| *Timing of marijuana use initiation* | | | | | | | |
| **ACE** | **.43 [.18, .71]** | **.36 [.09, .57]** | **.22 [.16, .30]** | **1108.79** | **1110.80** | **1087.82** | **-** |
| AE | .79 [.72, .84] | - | .21 [.16, .28] | 1112.96 | 1114.86 | 1094.17 | .01 |
| CE | - | .66 [.58, .73] | .34 [.27, .42] | 1118.18 | 1120.07 | 1099.39 | <.001 |
| E | - | - | 1.00 [1.00, 1.00] | 1232.54 | 1234.30 | 1215.91 | <.001 |

*Note:* AE and CE sub-model fitted equally for physical victimization, thus variance components for both sub-models were reported. For timing of alcohol and marijuana use initiation, variance components were estimated separately for males and females, with M = male, F = female, in subscripts of model names. Models in bold texts are best fitting models.

*Table S3.11.* Frequency of Participants Reporting Different Levels of Victimization

| **Form of victimization** | **Response = 1 (“Never”)** | | **Response = 2 or higher** | |
| --- | --- | --- | --- | --- |
|  | **Frequency** | **%** | **Frequency** | **%** |
| Physical victimization | 330 | 69.9 | 142 | 30.0 |
| Verbal victimization | 161 | 34.0 | 313 | 66.0 |
| Relational victimization | 222 | 46.9 | 251 | 53.0 |

*Note:* For each item of child self-reported victimization, 1 = Never, 2 = Rarely, 3 = Sometimes, 4 = Often, 5 = Always.
